# The differential impact of air pollution on insect chemical communication

**DOI:** 10.1038/s43247-026-03608-7

**Published:** 2026-05-16

**Authors:** Ben Langford, Jonathan Williams, Jérôme Casas

**Affiliations:** 1 UK Centre for Ecology and Hydrology, Bush Estate, Penicuik, UK; 2 Atmospheric Chemistry Department, Max Planck Institute for Chemistry, Mainz, Germany; 3 Climate and Atmosphere Research Center, The Cyprus Institute, Nicosia, Cyprus; 4 Insect Biology Research Institute IRBI, University of Tours, CNRS, UMR 7261, Tours, France

**Keywords:** Atmospheric chemistry, Ecosystem ecology

## Abstract

Airborne chemical communication is vital for insect survival and reproduction, yet its vulnerability to anthropogenic air pollution is poorly understood. We investigate how the physicochemical properties of alarm and sex pheromones influence their atmospheric persistence under pristine and polluted conditions. Using a volatility basis set framework, we show that alarm pheromones—small, volatile, and chemically simple—remain in the gas phase with lifetimes largely unaffected by pollution. In contrast, sex pheromones are larger, less volatile, and more reactive, suffering dramatic reductions in lifetime and spatial range under polluted nocturnal conditions. A key driver of this decline is gas-to-aerosol partitioning, which can remove pheromones from the gas phase faster than chemical degradation. While this may impair detection, aerosol-bound pheromones could be protected from oxidation, potentially extending their lifetime. These findings reveal that air pollution disproportionately disrupts sex pheromone signalling, highlighting a nuanced pathway by which ecological communication is impaired.

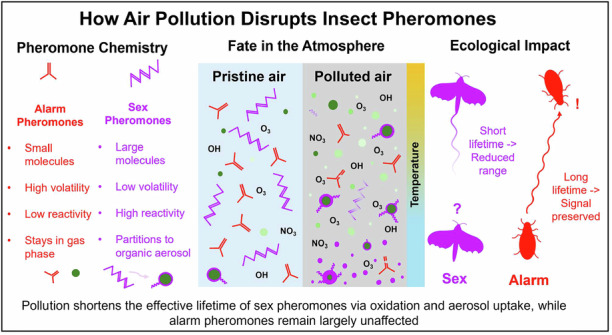

## Abstract (web summary)

Insect airborne communication, especially sex pheromone signaling, is vulnerable to anthropogenic air pollution, according to an integrated model of gas phase oxidation and gas-to-aerosol partitioning.

## Introduction

Pheromones are chemical signals that enable communication among insects, coordinating essential behaviours related to survival, reproduction, and social organisation^1–4^. These compounds vary in their chemical properties and volatility, and their release patterns are adapted to the specific ecological contexts in which they function^5^. They can be classified into various categories, including sex, aggregation and alarm signals, each serving a distinct communicative purpose^1^. The volatility of these compounds plays a critical role in determining their dispersal efficiency and the spatial scale over which messages function. Both volatility and rate of degradation through atmospheric oxidation are key factors influencing the endurance and overall effectiveness of these chemical signals.

Pheromones span a range of volatilities, from highly volatile organic compounds (VOCs), which evaporate quickly to enable rapid signal dispersal, to semi-volatile organic compounds (SVOCs), which are larger, less volatile and more chemically reactive in air. The volatility and atmospheric lifetime of these compounds against oxidation influence their effectiveness: VOCs disperse rapidly but fade quickly, while SVOCs, despite evaporating more slowly over extended periods, are often more susceptible to degradation due to their chemical structure, limiting their longevity once in the gas phase.

The transient nature of many pheromones ensures signals remain relevant and timely, preventing redundancy through persistence that could cause confusion. This variability in volatility and reactivity reflects the differing time and spatial scale of chemical communication, from immediate social interactions to longer-lasting environmental cues that influence group behaviour over longer ranges.

Anthropogenic disturbances, particularly air pollution, pose a growing threat to insect communication^6–10^. Insects evolved in relatively clean atmospheric conditions, like those still found in remote and undisturbed ecosystems such as the Amazon rainforest, where background ozone (O₃) levels can remain near 10 ppb, and aerosol concentrations are typically below 2 µg m⁻³. Today, elevated levels of atmospheric pollutants threaten to degrade the integrity and range of these chemical signals. Reactive compounds such as hydroxyl radicals (OH), O_3_ and nitrate radicals (NO_3_) can chemically alter pheromones, changing both their volatility, structure and atmospheric lifetime, rendering them unrecognisable to their target recipient^11–13^. In addition, aerosol pollution increases the surface area available for condensation, which may cause semi-volatile pheromones to transition from the gas phase to the aerosol phase. While the consequences of this phase shift for insect perception are not yet fully understood, it could influence the atmospheric persistence and spatial distribution of these signals.

Early studies raised concerns about the effects of air pollution on pheromone communication, notably Arndt^14^, who demonstrated that ozone degradation could disrupt aggregation signals in *Drosophila melanogaster*. More recent work by Jiang et al.^13^ has expanded these findings, showing that exposure to ozone (100 ppb) oxidises sex pheromones like cis-Vaccenyl Acetate and (Z)−7-Tricosene, significantly impairing mate attraction and disrupting normal mate recognition. These findings suggest that ozone-induced degradation may affect a broad range of species, undermining the effectiveness of pheromone-based communication^15,16^.

The degradation of pheromones via oxidation or ozonolysis reduces their efficacy, potentially impacting insect behaviour and communication. This is of particular concern given the vital role insects play in ecosystem functioning, including pollination, pest control and nutrient cycling. Disruptions to their communication systems could have profound ecological consequences, affecting biodiversity and the services that insects provide^6,11,17,18^.

Thousands of insect species across the globe rely on chemical signals for communication, deploying a vast array of compounds adapted to specific ecological contexts and behavioural strategies. However, the sheer diversity of these signals makes a comprehensive analysis infeasible. To address this, we focus on representative alarm and sex pheromones and examine their physicochemical behaviour under both pristine and polluted atmospheric conditions. Recognising the latitudinal variation in solar radiation, temperature, and pollution profiles, we simulate conditions broadly representative of day- and nighttime environments in (a) mid-latitude regions and (b) equatorial zones. This approach allows us to explore how volatility and atmospheric degradation might influence signal persistence and function across contrasting ecological settings.

Drawing inspiration from the volatility basis set (VBS) framework developed by Donahue et al.^19–21^, we examine the relationship between volatility (represented by C*) and the effective atmospheric lifetime of these signals. Alarm and sex pheromones were selected due to their contrasting roles and deployment strategies: alarm pheromones are typically used during the daytime by diurnal insects and are designed for rapid, short-range communication, while sex pheromones are predominantly released at night by nocturnal species and function over longer spatial and temporal scales. These differences make them ideal case studies for assessing how volatility and atmospheric degradation influence signal persistence and communicative function.

By investigating how changes in atmospheric conditions—such as variations in ozone levels and aerosol concentrations—affect the persistence of these key communication molecules, we aim to determine how air pollution impacts different classes of chemical signals. This research provides critical insights into how environmental stressors like air pollution can alter insect communication, with broader implications for insect behaviour, population dynamics and ecosystem services in the context of the polluted Anthropocene.

## Results

### Pheromones in volatility space

The pheromones selected for this study (Table S1) span a broad range of insect taxa (Hymenoptera, Coleoptera, Lepidoptera, Hemiptera, Isoptera, Acari and Diptera), functional groups and physicochemical properties—including volatility, molecular weight and reactivity—while representing two ecologically distinct signal types: alarm and sex pheromones. This diversity enables a generalised theoretical assessment of how air pollution might influence pheromone persistence and function in the atmosphere.

**Fig. 1:**
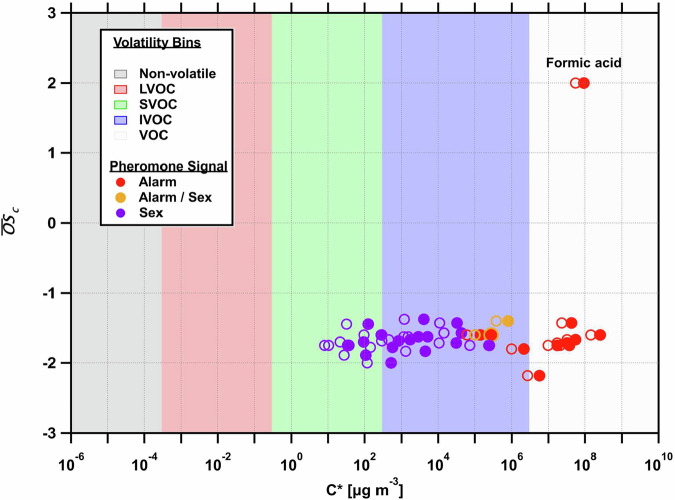
Volatility distributions of alarm and sex pheromones in VBS space. Common alarm and sex pheromones plotted in volatility basis set (VBS) space. Solid circles indicate data at 25 °C, while open circles represent data at 15 °C.

When these pheromones are plotted in volatility basis space, a clear separation emerges between the two pheromone signal classes. As shown in Fig. 1, most alarm pheromones fall within the volatile organic compound (VOC) bin (C* > 10⁶ μg m⁻³) at 25 °C, with a few transitioning into the intermediate-volatility organic compound (IVOC) range (10³ <C* ≤ 10⁶ μg m⁻³) at lower temperatures (15 °C). This indicates that alarm pheromones are highly volatile and are likely to remain entirely in the gas phase across a wide range of ambient temperatures and aerosol mass loadings.

In contrast, the sex pheromones display a broader range of volatility. At 25 °C, most fall into the IVOC range, but as temperature decreases, they shift markedly: at 15 °C, they are approximately evenly split between IVOC and semi-volatile organic compound (SVOC) bins (1 < C* ≤ 10³ μg m⁻³), and at 5 °C (see Fig. S1), nearly all occupy the SVOC space. This reflects their physicochemical characteristics: sex pheromones tend to have higher molecular weights and significantly lower saturation vapour pressures compared to alarm pheromones.

Both pheromone classes cluster within a narrow range of average carbon oxidation state values, typically between −2 and −1, consistent with direct emission by insects and minimal atmospheric oxidation. The one clear outlier is formic acid, which sits at an oxidation state of +2. This is expected, as formic acid is a single-carbon molecule fully functionalised with both a hydroxyl and a carbonyl group, resulting in a high degree of oxidation per carbon atom.

### Pheromone lifetime

**Fig. 2:**
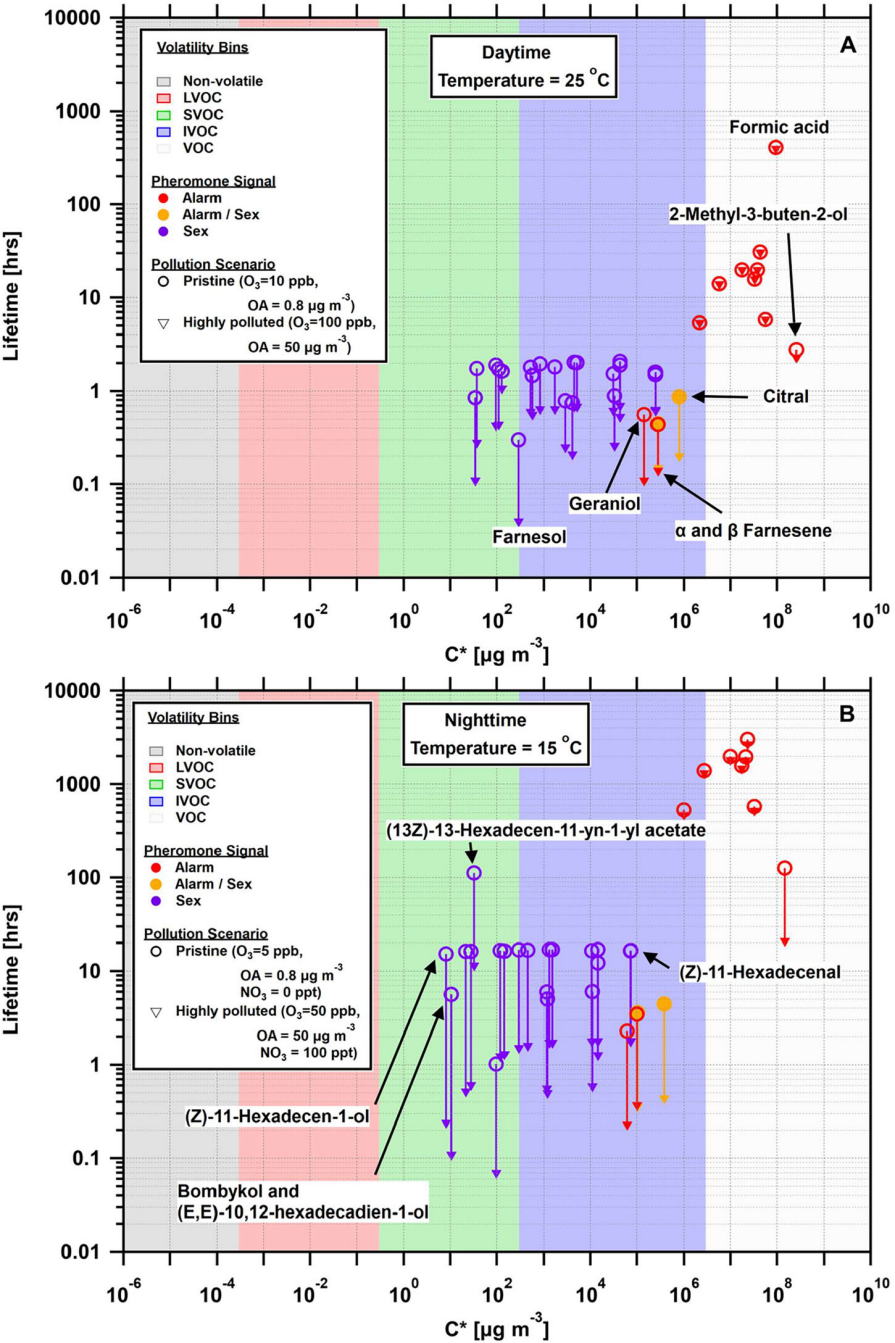
Pollution-driven divergence in alarm and sex pheromone lifetimes. Alarm and sex pheromones plotted in volatility basis set (VBS) space, using C* as an invariant volatility coordinate (defined at fixed temperature and γ₀). Atmospheric lifetime is shown on the y-axis. Open circles indicate lifetimes under pristine atmospheric conditions (Table 1), and arrows show the reduction in lifetime under highly polluted conditions. **A** represents a typical daytime scenario (25 °C) with daytime oxidant levels; **B** represents a nocturnal scenario (15 °C) with nighttime oxidant levels. The C* axis is used here to organise compounds along the volatility continuum and to illustrate how volatility and intrinsic chemical reactivity together drive the divergence in atmospheric lifetimes between alarm and sex pheromones.

Rather than plotting volatility basis against the traditional oxidation state, Fig. 2 (panels A and B) uses pheromone atmospheric lifetime (Eq. 13) as the y-axis variable. This substitution enhances the separation between pheromone classes. Panel A shows lifetimes for typical mid-latitude daytime temperatures. Alarm pheromones, which are typically used during daylight hours by diurnal insects to signal immediate threats, cluster at significantly longer lifetimes, with a median of 5.8 h, ranging from approximately 0.44 to over 400 h under daytime pristine conditions. These values extend further at night, ranging from 2.32 to 30,000 h, with a median of 582 h. As the lifetime due to chemical degradation increases, dry deposition—though typically slow—becomes increasingly important for determining signal longevity.

In contrast, sex pheromones—used predominantly by nocturnal insects during dusk and nighttime to facilitate mate attraction—exhibit much shorter lifetimes. While we report their daytime lifetimes (18 min to 2 h, median 1.6 h) for comparative purposes, it is their nighttime persistence that is biologically relevant^22^. Under pristine night-time conditions, sex pheromone lifetimes range from 1 to 112 h, with a median of 16 h. This distinction reflects fundamental differences in atmospheric persistence, driven by volatility and molecular structure of the two classes that align with their ecological function: short-range, fast-acting signals during the day versus longer-range, slower-dispersing signals at night

These distinctions become even more pronounced under highly polluted conditions (see Table 1). The arrows in Fig. 2 indicate the shift in lifetime for each pheromone when moving from pristine to highly polluted atmospheres. During the daytime, the lifetimes of sex pheromones decrease by almost one order of magnitude, falling to between 2.6 min and 1 h, with a median of 29 min. The impact is even more pronounced at night, where lifetime reductions can reach up to 1.5 orders of magnitude, with values dropping to a similar range as daytime polluted conditions, ~4 min and 11 h and a median of 0.92 h. Although the absolute values remain longer than during the day, this marked decline under polluted nighttime conditions is particularly concerning given the ecological role of sex pheromones in nocturnal communication.

**Table 1:** Environmental and atmospheric conditions used to define pollutant scenarios

	Pristine	Polluted	Highly Polluted
OA particle number count [#cm^−3^]	3000	30,000	50,000
OA diameter [nm]	75	110	120
OA density [g cm^−3^]	1.2	1.2	1.2
OA mass loading [μg m^−3^]	0.8	25.1	54.3
OH [molecules cm^−3^] (day/night)	1.5 × 10^6^/1.5 × 10^4^	1.5 × 10^6^/1.5 × 10^4^	1.5 × 10^6^/1.5 × 10^4^
Daytime O_3_ [ppb] (day/night)	10/5	50/25	100/10
NO_3_ [molecules cm^−3^] (day/night)	2.5 × 10^2^/2.5 × 10^4^	2.5 × 10^2^/1.28 × 10^6^	2.5 × 10^2^/2.5 × 10^6^
Temperature [°C] (day/night)	25/15	25/5	25/15

Summary of ground-level atmospheric conditions used to represent a range of environments from pristine to highly polluted. Parameters include organic aerosol (OA) properties (particle number concentration, size, density and mass), oxidant concentrations (OH, O₃ and NO₃) for day and night, and ambient temperature. These conditions form the basis for scenario-based analysis of pheromone fate and reactivity.

In stark contrast, the atmospheric lifetimes of alarm pheromones show minimal variation under polluted conditions, with a median of 5.84 h (ranging from 0.1 to 400 h) during the day and 525 h at night (ranging from 0.23 to 14,300 h), indicating strong resilience to anthropogenic pollution and reinforcing their classification as highly volatile, gas-phase stable compounds. The strong differential between median day and nighttime lifetimes demonstrates that for most alarm pheromones, the hydroxyl radical appears to be the dominant sink rather than anthropogenic oxidants (e.g. O_3_ and NO_3_).

Within this group, however, several notable exceptions exhibit significantly shorter atmospheric lifetimes, including citral, geraniol, (E)-β-farnesene and (E)-α-farnesene. Although these compounds are often discussed in the context of insect signalling—for example, citral and α-farnesene act as sex pheromones in some species (e.g. Pieris napi^23^) and as alarm signals in others (e.g. certain mites^24^)—they are also widely emitted as plant volatiles. Likewise, geraniol and β-farnesene, recognised alarm pheromones in sycamore lace bugs^25^ and aphids^26^, respectively, are also common floral and plant emissions. Their short atmospheric lifetimes are therefore consistent with their high chemical reactivity as biogenic VOCs, rather than being uniquely tied to their roles in insect communication. Because these compounds participate in multiple ecological interactions—including, in some cases, functioning as kairomones—their rapid degradation may help restrict long-range detectability while still enabling effective short-range signalling.

Repeating the analysis shown in Fig. 2 for temperatures more representative of equatorial climates (daytime 30 °C, nighttime 25 °C) showed a negligible change in alarm pheromone lifetimes under pristine conditions, and a modest increase in sex pheromone lifetimes (+4.2%). Under highly polluted conditions, the median alarm pheromone lifetime again remained stable, whereas sex pheromones exhibited a substantial increase of +39%, rising from 0.92 to 1.28 h. These findings suggest that while alarm pheromones are largely insensitive to temperature shifts, sex pheromones may persist slightly longer in warmer, polluted environments. A full set of results is shown for all temperature regimes in Table S2 and Fig. S2 of the Supplementary Information. The atmospheric lifetimes presented here represent e-folding times—the time required for a compound’s concentration to decrease to approximately 37% of its initial value due to chemical degradation. For spatial context, a typical nighttime windspeed of 1.5 m/s would allow a sex pheromone to travel approximately 6.5 km through a polluted atmosphere, during the median 1.2-h lifetime. In contrast, the more reactive pheromones, with lifetimes of ~4 min, would only travel about 360 m, underscoring their highly localised nature and the narrow window for effective communication. However, these distances reflect only the potential for chemical persistence. In practice, turbulent mixing and advection rapidly fragment odour plumes into intermittent filaments interspersed with clean air, reducing the frequency and intensity of detectable odour encounters^27,28^. Chemical degradation further accelerates this loss, shortening the lifetime and weakening the signal strength of the odour filaments that insects rely on for navigation.

### Loss to aerosols

#### Fraction partitioned to aerosols

**Fig. 3:**
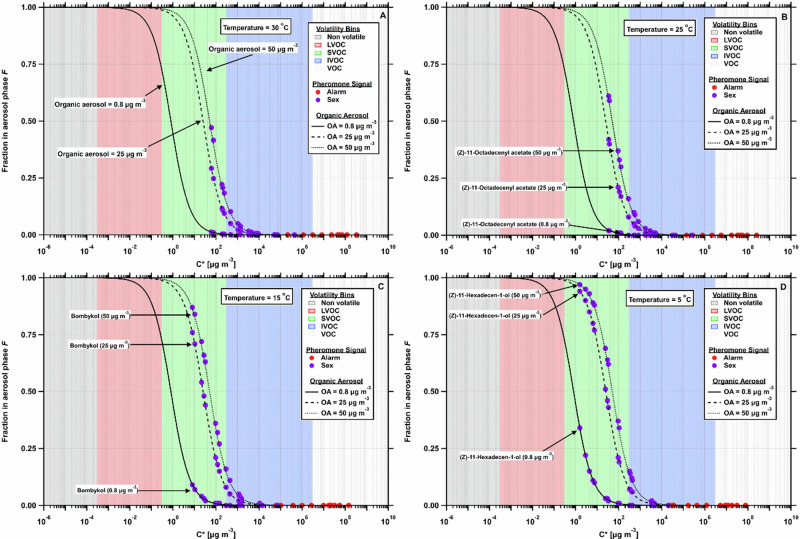
Pheromone partitioning between gas and aerosol phases across environments. Alarm and sex pheromones plotted in volatility basis set (VBS) space, with the aerosol-phase fraction (*F*) on the y-axis. Panels show temperatures of 30 °C (**A**), 25 °C (**B**), 15 °C (**C**) and 5 °C (**D**), for organic aerosol mass loadings of 0.8, 25 and 50 µg m⁻^3^. Because the aerosol-phase fraction is defined by *F* = 1/(1 + C*/*M*), C* serves as the volatility coordinate that determines how each compound partitions between gas and particle phases under different pollution levels.

The more dramatic relative decrease in sex pheromone lifetime at night is driven in part by increased NO₃ concentrations, which help offset the reduction in the two primary daytime oxidants, OH and O₃. However, the key driver appears to be the cooler nighttime temperature (15 °C), which lowers the effective saturation concentration and shifts more of the sex pheromones into the semi-volatile organic compound (SVOC) range. This shift increases partitioning to the aerosol phase, making aerosol uptake a more significant contributor to overall loss for semi-volatile pheromones. Consequently, under warmer climates, this effect is diminished, and lifetimes are less affected. Figure 3A shows the fraction of each pheromone, *F* (Eq. 4), that partitions to aerosol, plotted against the volatility basis set. The plots are shown for four temperatures, 30, 25, 15 and 5 °C, and each includes three pollution scenarios: pristine (0.8 μg m⁻³), moderately polluted (25 μg m⁻³), and highly polluted (50 μg m⁻³). From these plots, it is clear that, as temperature decreases, pheromone volatility also decreases. A significant proportion of the sex pheromones shift into the SVOC range, while the alarm pheromones remain within the VOC or IVOC range across all conditions. Under pristine conditions at 25 °C, nearly all sex pheromones remain entirely in the gas phase.

(Z)−11-Hexadecen-1-ol, bombykol, and (Z)−11-octadecenyl acetate were the three sex pheromones with the lowest saturation vapour concentrations. Under pristine daytime conditions (25 °C), the equilibrium model predicted that 2%, 2% and 1% of each compound, respectively, would partition into the aerosol phase. In a highly polluted scenario (e.g., OA = 50 μg m⁻³), these fractions increased dramatically to 59%, 61% and 37%, respectively.

At night, when a cooler temperature of 15 °C was assumed, the aerosol-phase fractions rose from 9%, 8% and 4% under pristine conditions to 87%, 84% and 72% under polluted conditions. At very cool temperatures (5 °C), over 50% of (Z)−11-hexadecen-1-ol partitioned to the aerosol phase even under pristine conditions, increasing to 95% in a highly polluted atmosphere.

Switching from the equilibrium model (Eq. 4) to the irreversible uptake model (Eq. 11) further increased the fraction of each compound partitioning to the aerosol phase. Under the low-temperature, high-pollution scenario, eight of the listed sex pheromones were predicted to be completely removed from the gas phase. Therefore, for sex pheromones that irreversibly partition to aerosol surfaces, the lifetimes presented in Fig. 2 would be even shorter. Full results from the irreversible uptake model are provided in the Supplementary Information in Figs. S3 to S6 and in Table S3.

Overall, the strong temperature dependence of sex pheromone volatility implies that gas-to-aerosol partitioning will have the greatest impact at higher latitudes, while its effect is reduced in the warmer equatorial regions.

#### Reduction in lifetime due to gas-to-aerosol partitioning

The characteristic timescale for aerosol uptake was calculated for each pheromone using Eq. 11, with values ranging from 17 to 47 min and an average of 39 min. This was comparable to the gas phase lifetime (e.g. OH, O_3_ and NO_3_) of some of the most reactive pheromones. Multiplying this timescale by *F* yields the lifetime of each pheromone with respect to aerosol-phase loss, as shown in Eq. 14. The percentage reduction in overall pheromone lifetime due to aerosol uptake is then calculated using Eq. 1:

1 $${Reduction}\,\left( \% \right)=\,\left(\frac{{{Lifetime}}_{{Gas}}-{{Lifetime}}_{{Gas}+{Aerosol}}}{{{Lifetime}}_{{Gas}}}\right)\times 100$$

**Fig. 4:**
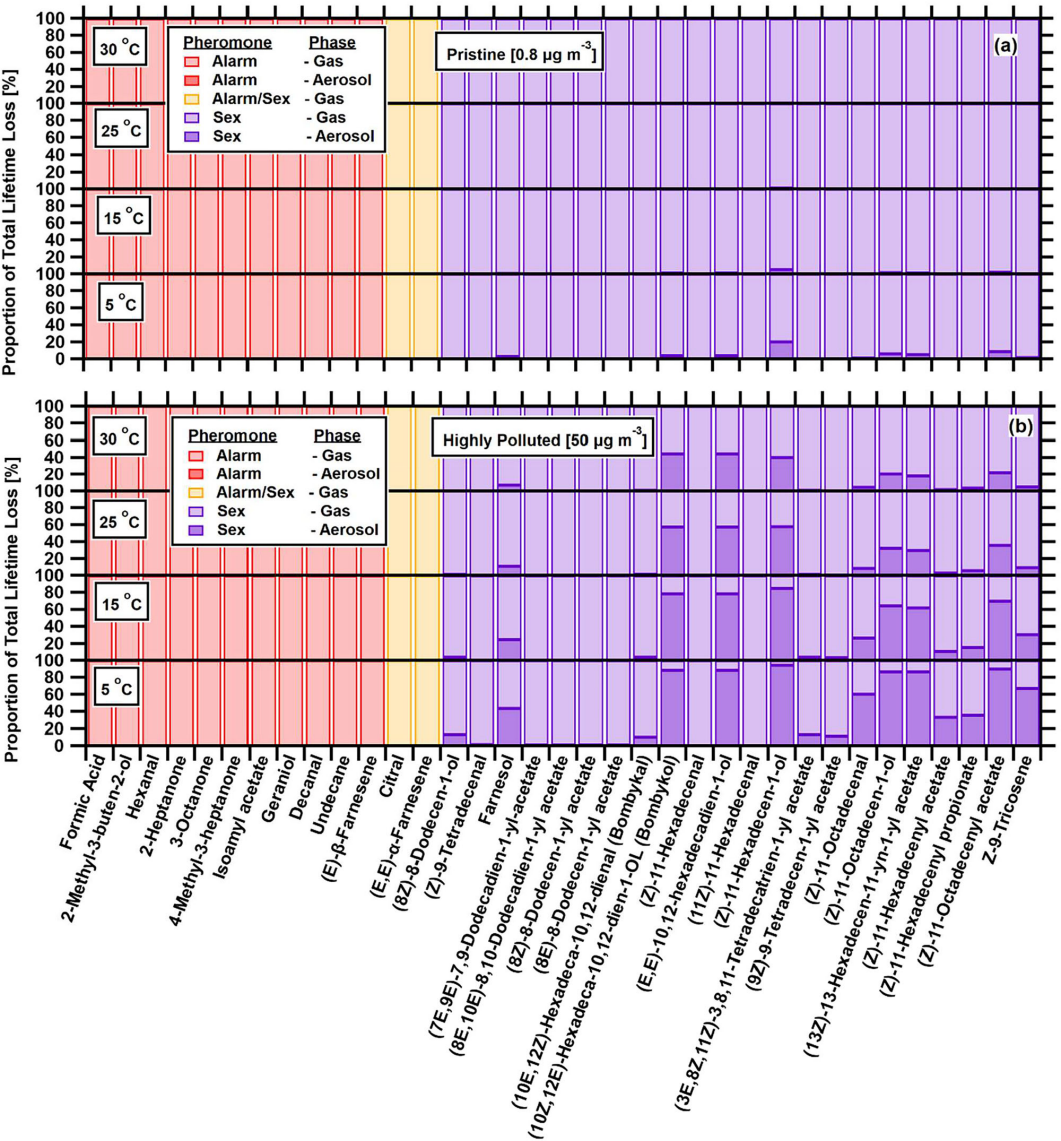
Reduction in pheromone lifetimes due to aerosol partitioning. Bar chart showing the percentage reduction in pheromone lifetime due to partitioning from the gas to the aerosol phase at temperatures of 30, 25, 15 and 5 °C. Pheromones are separated into Alarm (red) and sex (purple) classes, with light shades indicating pristine conditions (**a**) (organic aerosol = 0.8 μg m^−3^) and solid bars representing highly polluted conditions (**b**) (organic aerosol = 50 μg m^−3^).

Figure 4 presents this reduction for each compound listed in Table S1, across a range of temperatures and pollution scenarios. As already observed, alarm pheromones showed no partitioning to the aerosol phase under any of the conditions evaluated, and their lifetimes remain unchanged. In contrast, most sex pheromones exhibited increasing partitioning as temperature decreased. However, under pristine conditions, the reduction in lifetime remains minimal, typically less than 20% at 5 °C.

Under highly polluted scenarios, the percentage reduction in lifetime becomes more pronounced, reaching approximately 60% at 25 °C and exceeding 90% at 5 °C. The pheromones experiencing the greatest reductions include (10Z,12E)-Hexadeca-10,12-dien-1-ol (Bombykol), (E,E)−10,12-Hexadecadien-1-ol, (Z)−11-Hexadecen-1-ol, (Z)−11-Octadecen-1-ol, (13Z)−13-Hexadecen-11-yn-1-yl acetate, and (Z)−11-Octadecenyl acetate. These compounds have the lowest vapour pressures, making them more susceptible to aerosol-phase partitioning. Notably, several of the compounds with the highest degree of partitioning contain a terminal hydroxyl group, as indicated by the ‘1-ol’ suffix. The presence of this polar functional group likely enhances their affinity for the aerosol phase through hydrogen bonding and reduced volatility, contributing to their greater loss from the gas phase.

Several sex pheromones show no significant tendency to partition to the aerosol phase, even at low temperatures. These include the Dodecadien-1-yl acetate isomers, the 8-Dodecen-1-yl acetate isomers, and (11Z)−11-Hexadecenal, all of which tend to remain within the IVOC volatility range, even at very low temperatures. Notably, these compounds are either esters or aldehydes, functional groups that are generally less polar and less prone to hydrogen bonding than alcohols. This likely contributes to their limited interaction with the aerosol phase and their tendency to remain in the gas phase under all conditions tested.

## Discussion

Our theoretical analysis reveals that specific classes of insect pheromones are differentially affected by anthropogenic air pollutants, with important implications for their ecological function. Alarm pheromones are typically small, low molecular weight compounds with high volatility. These properties ensure rapid release and dispersion—key features for their role in triggering immediate, short-range behavioural responses. Despite their volatility, alarm pheromones often exhibit long atmospheric lifetimes, ranging from 6 to over 350 h, due to their chemical simplicity and low reactivity with atmospheric oxidants. While this extended lifetime may be ecologically redundant, the high volatility ensures signals are released into the gas phase efficiently and reach their recipient quickly, allowing for evasive action to be taken.

However, notable exceptions exist. Certain alarm pheromones, such as geraniol and (E)-β-farnesene, exhibit unusually short atmospheric lifetimes, likely due to their multiple double bonds. These compounds are also known to function as kairomones, chemical cues that can be exploited by predators. In such cases, rapid degradation may be selectively advantageous, reducing the risk of predator detection while still fulfilling their communicative function. These exceptions highlight the nuanced evolutionary trade-offs shaping pheromone chemistry in different ecological contexts.

In contrast, sex pheromones tend to be larger, more complex molecules, often containing multiple double bonds and functional groups that make them highly susceptible to oxidation by OH, O₃, and NO₃. Their higher molecular weight also reduces their volatility, increasing the likelihood of gas-to-aerosol partitioning. These compounds typically have much shorter atmospheric lifetimes, which aligns with their function as specific, targeted signals meant to be detected over moderate distances and within narrow time windows.

However, our analysis is theoretical and based on a macro-scale equilibrium framework, rather than detailed molecular simulations, and is subject to several limitations. Many of the pheromones considered lack experimentally determined physicochemical properties or atmospheric degradation rates, requiring us to rely on predictions based on structure–activity relationships. While these tools provide valuable first-order estimates, they carry inherent uncertainties^29^. For example, a sensitivity analysis comparing lifetimes derived using structural activity relationships versus those based on literature values suggested lifetimes to be overestimated by an average of 27% (Fig. S8 of the SI). Similarly, our use of C* and simplistic characterisation of organic aerosol properties offers a useful generalisation but does not account for compound-specific interactions with aerosol constituents or dynamic environmental conditions. As such, our results should be viewed as a conceptual framework rather than definitive predictions. They cannot capture the extraordinary diversity of insect species, each with unique ecological niches and metabolic strategies. Exceptions to the broad patterns we describe are numerous: some moths call during daylight^30^, others expel aerosols immediately after release^31^, and calling behaviour ranges from exposed habitats to dense vegetation. These examples underscore that while our framework identifies general trends, real-world signalling strategies are far more heterogeneous. Empirical studies are now needed to test these hypotheses under controlled and field conditions, including direct measurements of pheromone lifetimes, partitioning behaviour, and insect responses in polluted environments. Bridging this gap between theory and observation will be essential to assess the ecological relevance of atmospheric degradation and partitioning processes, and to understand their potential role in the broader decline of insect populations and the ecosystems they support. A promising starting point for such empirical work is to investigate what we identify as a key and previously underappreciated finding of this study: the significant role of gas-to-aerosol partitioning in reducing the effective lifetime of certain sex pheromones.

While oxidation has long been recognised as a major loss pathway, our results show that under polluted conditions, especially at lower ambient temperatures, a substantial fraction of these semi-volatile compounds partitions into the aerosol phase on time scales similar to or shorter than reactions with gas phase oxidants. This phase transition does not reduce atmospheric lifetime in the conventional chemical sense, but it does alter the physical state and potentially the bioavailability of the signal. For example, semi-volatile pesticides that partition to aerosols have been shown to exhibit extended lifetimes with respect to gas-phase oxidants^32^. By analogy, we note the speculative possibility that semi-volatile pheromones may gain a similar degree of protection once partitioned to aerosol, thereby preserving their chemical integrity and extending their detectable lifetime. Although it remains uncertain whether insects can perceive pheromones in the aerosol phase^33^, the altered transport dynamics and potential for delayed re-volatilisation could still influence signal dispersion and reception. Thus, rather than simply representing a loss mechanism, aerosol partitioning may act as a reservoir, modulating both the spatial and temporal characteristics of pheromone-mediated communication in polluted environments. The result is a dramatic reduction in the functional lifetime of gas-phase sex pheromones in polluted environments, potentially by orders of magnitude compared to pristine conditions. This may have profound implications for insect communication, particularly for species that rely on long-range chemical signalling for mate location. As atmospheric pollution and climate change continue to alter the chemical landscape, the disruption of pheromone signalling could contribute to declines in insect populations and biodiversity, with cascading effects on the ecosystems that depend on them.

## Materials and methods

### Gas phase degradation

We quantified the atmospheric persistence of each pheromone by calculating its gas-phase lifetime from its reactivity with major atmospheric oxidants and its susceptibility to physical loss. During the daytime, the hydroxyl radical (OH) and ozone are the primary oxidants responsible for VOC degradation^34^. At night, the nitrate radical becomes the dominant oxidant, forming in reactions between nitrogen oxides and ozone in the absence of sunlight. The nitrate radical efficiently reacts with unsaturated VOCs, though its rapid photolysis during the day limits its relevance to nocturnal chemistry ^35^.

The rate of reaction for a VOC with each oxidant can be described by a rate constant, which quantifies how quickly the reaction occurs under defined conditions of temperature and pressure. For a given VOC, the rate constants associated with its reactions with OH, O_3_ and NO_3_ — denoted as *k*_*OH*_, *k*_*O3*_ and *k*_*NO3*_ (cm^3^ molecule^−1^ s^−1^) are used in combination with the respective oxidant concentrations to calculate the atmospheric lifetime (${\tau }_{{gas}}$) of the compound. The lifetime is expressed as:

2 $${\tau }_{{gas}}=\frac{1}{\left({k}_{{OH}}\cdot \left[{OH}\right]\right)+\left({k}_{{O}_{3}}\cdot \left[{O}_{3}\right]\right)+\left({k}_{{{NO}}_{3}}\cdot \left[{{NO}}_{3}\right]\right)}$$

where [OH], [O_3_] and [NO_3_] are the concentrations of hydroxyl radicals, ozone and nitrate radicals, respectively. The summation of the products of rate constants and oxidant concentrations accounts for the integrated reactivity of the VOC with the oxidants present in the atmosphere.

### Rate constants for OH, O_3_ and NO_3_

Table S1 lists the insect pheromones selected for this study, along with rate constants for their reactions with OH, O₃ and NO₃. Where experimental data were available, rate constants were directly obtained from the literature. However, for many of the pheromones included in this study, direct experimental rate constants were not available. In these instances, rate constants were derived using structure-activity relationships (SARs), a widely accepted method for estimating reaction rates based on the chemical structure of the compounds.

Episuite, a software tool developed by the US EPA for predicting atmospheric fate and transport properties, was employed to estimate rate constants for reactions with OH and O₃. Specifically, the rate constants were derived using SAR-based models that relate the chemical structure of each pheromone to its reactivity with these atmospheric species. For OH rate constants, Episuite uses an empirical SAR model that incorporates parameters such as the presence of functional groups (e.g. double bonds, hydroxyl groups) and the overall molecular size and structure. Similarly, for O₃ reactions, where literature values were unavailable, rate constants were predicted using an O₃-specific SAR model that accounts for factors like conjugation and the potential for ozonolysis reactions. These models allow for the estimation of reactivity even in the absence of direct experimental data.

When calculating ${\tau }_{{gas}}$ for each pheromone, OH concentrations were set to 1.5 × 10^6^ molecules cm^−3^ for daytime conditions and 1.5 × 10^4^ molecules cm^−3^ was used for the night. This reflects the shutoff of OH production at night and its rapid decay. OH is not zero at night because reactions of O_3_ with some double-bonded molecules generate OH.

Ozone concentrations were varied to reflect pristine (10 ppb), moderate (50 ppb) and highly polluted scenarios (100 ppb), equivalent to ~2.49 × 10^11^, 1.24 × 10^12^ and 2.47 × 10^12^ molecules cm^−3^, respectively. At night, O_3_ concentrations are also typically low due to the shutoff of photochemical production and titration by nitric oxide from the soil in pristine environments, but the decline is not as dramatic as the reduction in OH. Here, we set nighttime concentrations to 50% of the daytime values.

For many of the pheromones used in this study, rate constants with respect to NO_3_ were not available in the literature. Instead, they were calculated using the Generator for Explicit Chemistry and Kinetics of Organics in the Atmosphere (GECKO-A) (https://geckoa.lisa.u-pec.fr/). NO_3_ concentrations were set to 2.5 × 10^2^ molecules cm^−3^ during daytime and 2.5 × 10^4^ molecules cm^−3^ at night when simulating pristine conditions. For the highly polluted scenario, nighttime concentrations were increased to 2.5 × 10^6^ molecules cm^−3^.

### Saturation concentration (C*) and partitioning

In addition to gas phase oxidation, insect pheromones can transition from the gas phase via partitioning to aerosols. The Volatility Basis Set (VBS) provides a conceptual framework for categorising organic compounds, including pheromones, based on their effective saturation concentrations (C*), a parameter crucial to understanding gas–aerosol partitioning behaviour in the atmosphere^20,21^. The value of C* represents the concentration at which a compound would theoretically partition equally between the gas and aerosol phases under conditions with negligible absorbing aerosol mass (*M*). As a property intrinsic to the compound, C* reflects its volatility and thermodynamic equilibrium behaviour, serving as a foundational metric for characterising secondary organic aerosol (SOA) formation processes.

The saturation concentration, typically expressed as μg m^3^, can be calculated as

3 $${C}^{* }=\frac{{M}_{w}{P}_{{vap}}{y}_{0}}{R\,T}\cdot {10}^{6}$$

where ${M}_{w}$ is the molecular weight of the compound (g mol^−1^), ${P}_{{vap}}$ is the vapour pressure at 25 °C (Pa), *R* is the universal gas constant (J mol^−1^ K^−1^), T is the temperature (K), 10^6^ is the necessary conversion factor to obtain units of μg m^3^ and ${y}_{0}$ is an activity coefficient. For simplicity, we set *y*_*0*_ to 1 for all molecules, recognising that this may overestimate C* for larger, less-ideal species; a sensitivity analysis exploring the influence of *y*_*0*_ is provided in the Supplementary Information. Organic compounds are traditionally classified within the VBS framework into several volatility regimes based on their C* values. Non-volatile organic compounds (NVOCs), with C* ≤ 10^−3^ μg m^3^, reside almost entirely in the aerosol phase under all atmospheric conditions. Low-volatility organic compounds (LVOCs), defined by 10^−3^ < C* ≤ 1 μg m^3^, predominantly partition into the aerosol phase, even at low aerosol loadings. Semi-volatile organic compounds (SVOCs), spanning 1 < C* ≤ 10^3^ μg m^3^, exhibit partitioning behaviour highly sensitive to aerosol loading, existing in both gas and aerosol phases depending on the availability of absorbing mass. Intermediate volatility organic compounds (IVOCs), characterised by 10^3^ < C* ≤ 10^6^ μg m^3^, remain primarily in the gas phase, though their oxidation products may contribute significantly to SOA formation. Volatile organic compounds (VOCs), with C* > 10^6^ μg m^3^, exist almost exclusively in the gas phase. Traditionally, the volatility basis set is represented as a two-dimensional space, with C* on the *x*-axis and oxidation state $({{OS}}_{c})$ on the *y*-axis. Where the oxidation state is calculated based on the ratio of oxygen and hydrogen to carbon, as:

4 $${{\overline{{OS}}_{c}}}=2\; {{\rm{x}}}\; ({{\rm{O}}}:{{\rm{C}}}){{\mbox{-}}}({{\rm{H}}}:{{\rm{C}}})$$

As gas phase organic molecules become increasingly oxidised, their C* decreases, and they traverse towards the top left of the volatility space, eventually partitioning into the aerosol phase.

The introduction of absorbing organic aerosol into the volatility basis set (*M*) modifies the equilibrium partitioning of semi-volatile species, shifting their distribution between the gas and aerosol phases. The fraction of a compound in the aerosol phase (*F*) can be described mathematically by the expression^36^

5 $$F=\,\frac{1}{1+\frac{{C}^{* }}{M}}$$

This relationship reveals the dynamic interplay between a compound’s intrinsic volatility (C*) and the external conditions provided by the organic aerosol (*M*). When C* = *M*, the compound partitions equally between the phases (*F* = 0.5). As *M* increases beyond C*, the compound increasingly partitions into the aerosol phase, approaching *F* = 1. Conversely, when *M* is much smaller than C*, the compound predominantly resides in the gas phase, with *F* approaching zero. In this formulation, partitioning is treated as an equilibrium process, meaning that both condensation and desorption are implicitly balanced. While previous work (e.g. Jami et al.^37^) has shown that compounds like bombykol can rapidly partition to pure water aerosol but also desorb quickly due to insufficient binding (low K_bt), many pheromones possess higher octanol–air partition coefficients (*K*ₒₐ), suggesting stronger retention in organic aerosol and slower desorption. Therefore, we consider only the organic fraction of the total aerosol and assume *M* = 0.8 μg m⁻³ for pristine conditions representative of the remote Amazon^38^, *M* = 25 μg m⁻³ for moderately polluted conditions characteristic of rural locations downwind of urban areas^39^, and *M* = 50 μg m⁻³ for highly polluted conditions typical of severely impacted urban regions ^40^.

### Rate of gas-to-aerosol partitioning

Gas-to-aerosol partitioning is a key atmospheric process governing the fate of semi-volatile organic compounds (SVOCs), including insect pheromones. For compounds with sufficiently low volatility (i.e. low saturation concentration, C*), partitioning to organic aerosols can occur quickly^37^. However, this process is complex and highly dependent on both physicochemical properties and ambient aerosol characteristics.

To characterise the rate of partitioning, we employed a mass-transfer framework based on kinetic gas theory, modified using the Fuchs–Sutugin correction to account for non-continuum effects in the transition regime. The net rate constant for partitioning to aerosol particles (*k*′, s^−1^) is given by:

6 $${k}^{{\prime} }=\frac{\alpha \cdot c\cdot {A}_{{eff}}}{4}$$

where α is the mass accommodation coefficient (dimensionless, set at 0.5, with sensitivity study shown in the SI), c is the mean thermal velocity of the gas-phase molecule (m s^−1^), and ${A}_{{eff}}$ is the effective aerosol surface area concentration (m² m⁻³), modified by the Fuchs–Sutugin correction factor (*β*). This correction accounts for the finite mean free path of molecules and becomes particularly important under low-pressure or low-particle-size conditions.

The thermal velocity c is defined as:

7 $$c=\sqrt{\frac{8{RT}}{\pi {M}_{w}}}$$

where R is the universal gas constant (8.314 J mol^−1^ K^−1^), *T* is the absolute temperature (K), and *M*_*w*_ is the molar mass of the compound (kg mol^−1^). The effective surface area ${A}_{{eff}}$ is derived as:

8 $${A}_{{eff}}=\,\beta \cdot A$$

with *A* representing the geometric surface area of aerosols per unit volume, and *β* calculated using the Fuchs–Sutugin approximation:

9 $$\beta =\frac{1+{K}_{n}}{1+\left(1+0.377{K}_{n}+\frac{1.33{K}_{n}\left({K}_{n}+1\right)}{\alpha }\right)}$$

The Knudsen number, *Kn*, characterises the regime of gas–aerosol interactions and is typically calculated as the ratio of the mean free path of gas-phase molecules, *λ*, to the aerosol radius, *r*. In this study, due to the limited availability of accurate molecular diffusivities for all compounds considered, a simplified approach was adopted. The mean free path of air at 298 K was assumed to be *λ* = 0.066 μm, corresponding to standard atmospheric conditions (1 atm, 298 K). The Knudsen number was then estimated using Eq. 10:

10 $${K}_{{{n}}}=\frac{2\lambda }{{d}_{{{p}}}}$$

where *d*_*p*_ is the aerosol diameter in micrometres. For example, a 100 nm aerosol yields *K*_*n*_ = 1.32, placing the system within the transition regime between free molecular and continuum flow. In this regime, the Fuchs–Sutugin correction becomes necessary to accurately account for non-continuum effects on gas uptake by aerosols.

Using this framework, the characteristic timescale for aerosol uptake (${\tau }_{{part}}$, *s*) is obtained as:

11 $${\tau }_{{part}}=\frac{1}{{k}^{{\prime} }}$$

This timescale reflects the physical transfer of molecules to the aerosol surface but does not, in itself, determine whether the compound remains there. To assess the fraction of compound that is effectively taken up into the aerosol phase, we consider thermodynamic partitioning, typically modelled using absorptive partitioning theory shown in Eq. 5. This approach assumes reversible equilibrium between the gas and condensed phases, governed by a Raoult’s law-like relationship—in which the gas-phase concentration of a compound is reduced in proportion to its effective solubility in the condensed phase. In this framework, semi-volatile compounds are assumed to mix into the bulk organic phase of the aerosol, consistent with volatility basis set formulations. However, this assumption may not hold for biologically rare or chemically distinctive compounds such as insect pheromones, which are unlikely to have preexisting analogues within the aerosol matrix. In such cases, uptake may occur via surface adsorption, entrapment, or chemical reaction, and may be effectively irreversible. Once adsorbed, such compounds may not readily re-partition back to the gas phase, particularly if they undergo strong binding or transformation within the aerosol^37^. To explore this behaviour, we considered an alternative formulation in which partitioning is effectively irreversible, and *F* is defined as:

12 $$F=\min \left(1,\frac{M}{{C}^{* }}\right)$$

This capped expression ensures that *F* does not exceed unity, while allowing full uptake under sufficiently high aerosol loading.

In both formulations, temperature plays a central role in determining the extent and rate of partitioning. Lower temperatures reduce C*, increasing the aerosol-phase fraction (*F*), while simultaneously decreasing thermal velocity (*c*), thereby lengthening ${\tau }_{{part}}$. To explore this temperature sensitivity, saturation concentrations were recalculated from a reference value at 298 K (25 °C), representative of mid-latitude daytime conditions, to cooler nighttime conditions (e.g. 288 K, or 15 °C) using the Clausius–Clapeyron equation:

13 $${p}_{{vap}\,({night})}={P}_{{vap}({day})}\cdot \exp \left(-\frac{{\Delta H}_{{vap}}}{R}\cdot \left(\frac{1}{{T}_{{night}}}-\frac{1}{{T}_{{day}}}\right)\right)$$

where ${\Delta H}_{{vap}}$ is the enthalpy of vaporisation (J mol^−1^). This adjustment enables consistent comparison between day and night partitioning regimes and facilitates estimation of temperature-driven changes in *F* and ${\tau }_{{part}}$. The same approach was used when simulating the effects for conditions broadly representative of equatorial regions, where daytime temperatures were set to 30 °C and nighttime values to 25 °C.

Ultimately, the full impact of gas-to-aerosol partitioning on pheromone lifetimes in the atmosphere depends on both the rate of mass transfer (*k*′) and the effective uptake fraction, *F*. These values feed directly into the combined lifetime formulation described in Section “Combined lifetime (oxidation and partitioning)”.

### Combined lifetime (oxidation and partitioning)

To quantify the combined lifetime of gas-phase oxidation and gas-to-aerosol partitioning, an integrated model was developed, based on the contributions of each phase to the overall rate of loss.

The combined lifetime $({\tau }_{{total}})$ was calculated using the expression^32^

14 $${\tau }_{{total}}=\left(1-F\right)\cdot {\tau }_{{gas}}+F\cdot {\tau }_{{part}}$$

where ${\tau }_{{gas}}$ represents the gas-phase lifetime, governed by the chemical reactivity of the pheromone with atmospheric oxidants, ${\tau }_{{part}}$ corresponds to the characteristic timescale for gas-to-aerosol partitioning, and *F* is the fraction of the compound partitioned into the aerosol phase. The parameter *F* was determined from Eq. 4 under assumptions of equilibrium and from Eq. 11, when assuming irreversible uptake, using the saturation concentration (C*) and absorbing aerosol mass (*M*).

This formulation accounts for the relative contributions of gas-phase and aerosol-phase processes. The term $\left(1-F\right)\cdot {\tau }_{{gas}}$ quantifies the lifetime of the fraction of the VOC remaining in the gas phase, with degradation governed by reaction kinetics with hydroxyl radicals, ozone, and nitrate radicals, as described in Section “Gas phase Degradation”. In contrast, $F\cdot {\tau }_{{part}}$ reflects the shorter aerosol-phase timescale, determined by the efficiency of gas-to-aerosol partitioning processes. This shorter timescale results from rapid equilibrium established between the phases, driven by the mass accommodation coefficient (*α*), aerosol surface area (*A*), and temperature effects, as detailed in Section “Rate of Gas-to-Aerosol Partitioning”.

The relative contributions of gas-phase and aerosol-phase mechanisms are highly sensitive to environmental conditions, particularly aerosol mass concentrations, oxidant levels, and temperature variations. Higher aerosol concentrations increase *F*, shifting the lifetime toward faster removal in the aerosol phase. Conversely, when aerosol concentrations are low, *F* decreases, leaving the gas-phase oxidation as the dominant loss pathway. Temperature influences both *F* and the timescales involved, with lower temperatures reducing C* and accelerating partitioning dynamics while slowing gas-phase reactions. This sensitivity to external factors highlights the complex interplay between the two pathways and may lead to different dominant mechanisms in different regions, such as temperate versus equatorial climates.

### Atmospheric conditions—From pristine to highly polluted

Table 1 summarises the atmospheric conditions used to represent a range of environments, from pristine rainforest settings to highly polluted areas. These include typical values for organic aerosol (OA) particle number concentrations and sizes, as well as OA mass concentrations. In addition, oxidant concentrations for hydroxyl radicals (OH), ozone (O₃) and nitrate radicals (NO₃) are provided for both day- and night-time conditions, along with ambient temperature profiles. Nighttime ozone concentrations are set to 50% of daytime values, with the exception of the highly polluted scenario (City), where increased titration of O_3_ by NOx is expected. In this case, nocturnal ozone is set to 10% of daytime concentrations. While our analysis broadly distinguishes temperate and equatorial climates based on temperature, we do not apply a similar broad categorisation to OH concentrations, which are expected to be higher in equatorial regions.

## Supplementary information

Transparent Peer Review File

Supplementary Material

## Data Availability

All data is contained within the Supplementary Information, and the data supporting each figure are available from Figshare: 10.6084/m9.figshare.31900573.

## Code availability

The calculations underlying this study were implemented in a structured Microsoft Excel spreadsheet that accompanies the deposited data. The spreadsheet, which contains the pheromone lifetime calculations for the different pollutant scenarios and temperature (daytime and nighttime) conditions explored in the paper, is openly available via Figshare at 10.6084/m9.figshare.31900573.
