# Supplementary material for: The differential impact of air pollution on insect chemical communication
Source: Commun Earth Environ. 2026 May 16;7(1):631. doi: 10.1038/s43247-026-03608-7 (PMC13433313; doi:10.1038/s43247-026-03608-7)
Supplement: Supplementary file 1 [file 43247_2026_3608_MOESM1_ESM.pdf]

nature portfolio

Peer Review File

The Differential Impact of Air Pollution on Insect Chemical Communication

Corresponding Author: Dr Ben Langford

This file contains all editorial decision letters in order by version, followed by all author rebuttals in order by version.

Version 0:

Decision Letter:

** Please ensure you delete the link to your author home page in this e-mail if you wish to forward it to your coauthors **

Dear Dr Langford,

Please accept our apologies for the delay in sending a decision on your manuscript. Your manuscript titled "The Differential Impact of Air Pollution on Insect Chemical Communication" has now been seen by 3 reviewers, and we include their comments at the end of this message. They find your work of interest, but some important points are raised. We are interested in the possibility of publishing your study in Communications Earth & Environment, but would like to consider your responses to these concerns and assess a revised manuscript before we make a final decision on publication.

We therefore invite you to revise and resubmit your manuscript, along with a point-by-point response that takes into account the points raised. Please highlight all changes in the manuscript text file.

Please submit your point-by-point responses as a separate file, distinct from your cover letter where you can add responses to the Editors' comments that you do not want to be made available to the reviewers. Word files are preferred. We recommend that any figures, tables or graphs that are included in the response to reviewers are also included in the main article or Supplementary Information.

We are committed to providing a fair and constructive peer-review process. Please don't hesitate to contact us if you wish to discuss the revision in more detail.

Please use the following link to submit your revised manuscript, point-by-point response to the referees' comments (which should be in a separate document to any cover letter), a tracked-changes version of the manuscript (as a PDF file) and the completed checklist:
Link Redacted
** This url links to your confidential home page and associated information about manuscripts you may have submitted or be reviewing for us. If you wish to forward this email to co-authors, please delete the link to your homepage first **

We hope to receive your revised paper within six weeks; please let us know if you aren't able to submit it within this time so that we can discuss how best to proceed. If we don't hear from you, and the revision process takes significantly longer, we may close your file. In this event, we will still be happy to reconsider your paper at a later date, as long as nothing similar has been accepted for publication at Communications Earth & Environment or published elsewhere in the meantime.

Please do not hesitate to contact us if you have any questions or would like to discuss these revisions further. We look forward to seeing the revised manuscript and thank you for the opportunity to review your work.

Best regards,

Kalliopi Violaki, PhD
Editorial Board Member
Communications Earth & Environment
orcid.org/0000-0003-4612-3973

Alice Drinkwater, PhD
Associate Editor
Communications Earth & Environment
Consulting Editor
Communications Sustainability

EDITORIAL POLICIES AND FORMATTING

We ask that you ensure your manuscript complies with our editorial policies. Please ensure that the following formatting requirements are met, and any checklist relevant to your research is completed and uploaded as a Related Manuscript file type with the revised article.

For Manuscripts that fall into the following fields:
• Behavioural and social science
• Ecological, evolutionary & environmental sciences
• Life sciences
An updated and completed version of our Reporting Summary must be uploaded with the revised manuscript
You can download the form here:
https://www.nature.com/documents/nr-reporting-summary.pdf

Furthermore, please align your manuscript with our format requirements, which are summarized on the following checklist: Communications Earth & Environment formatting checklist

and also in our style and formatting guide Communications Earth & Environment formatting guide .

*** DATA: Communications Earth & Environment endorses the principles of the Enabling FAIR data project (http://www.copdess.org/enabling-fair-data-project/). We ask authors to make the data that support their conclusions available in permanent, publically accessible data repositories. (Please contact the editor if you are unable to make your data available).

All Communications Earth & Environment manuscripts must include a section titled "Data Availability" at the end of the Methods section or main text (if no Methods). More information on this policy, is available at http://www.nature.com/authors/policies/data/data-availability-statements-data-citations.pdf.

In particular, the Data availability statement should include:
- Unique identifiers (such as DOIs and hyperlinks for datasets in public repositories)
- Accession codes where appropriate
- If applicable, a statement regarding data available with restrictions
- If a dataset has a Digital Object Identifier (DOI) as its unique identifier, we strongly encourage including this in the Reference list and citing the dataset in the Data Availability Statement.

DATA SOURCES: All new data associated with the paper should be placed in a persistent repository where they can be freely and enduringly accessed. We recommend submitting the data to discipline-specific, community-recognized repositories, where possible and a list of recommended repositories is provided at http://www.nature.com/sdata/policies/repositories.

If a community resource is unavailable, data can be submitted to generalist repositories such as figshare or Dryad Digital Repository. Please provide a unique identifier for the data (for example a DOI or a permanent URL) in the data availability statement, if possible. If the repository does not provide identifiers, we encourage authors to supply the search terms that will return the data. For data that have been obtained from publically available sources, please provide a URL and the specific data product name in the data availability statement. Data with a DOI should be further cited in the methods reference section.

Please refer to our data policies at http://www.nature.com/authors/policies/availability.html.

REVIEWER COMMENTS:

Reviewer #1 (Remarks to the Author):

Let me start with a comment on the review process itself. Interdisciplinary papers such as this are possible because of the collaborative interactions of the co-authors, each of whom brings disciplinary excellence. As a reviewer, however, I work alone and cannot hope to match the breadth of the full complement of authors. Many of this paper’s methods and results reflect the expertise in chemistry that at least one of the authors brings. I do not share this skill set and will focus on the

biology under discussion. I hope the editors have suitable chemists for these portions of the manuscript. I will say that they did an excellent job of explaining their chemical approaches so that this Biologist could follow their logic, despite my inability evaluate critically.

With that said, this paper tackles an exciting topic and contributes to our understanding of volatile signaling among insects. Even though it depends on chemical techniques that I cannot evaluate critically, it is clear that the fundamental idea, that structural similarities among pheromones with different functions lead to similarities in chemical fates that may lead to similarities in how oxidizing pollutants such as O3 or NO3 radical affect their lifetimes and thus potential effectiveness as signals, is one that deserves serious study. This study sets out a framework for conducting such studies and, as such, has an important place in the literature.

The fundamental conclusion of the paper, that chemical structure patterns are shared within function and differ across functions in such a way that oxidant impacts differ across functions makes intuitive sense, but the detailed and explicit chemical approach laid out in the Methods section offers a way to quantitatively evaluate its significance. This explicit quantitation is one of the main values of the manuscript and to, to me, is a strong argument for its publication.

My impression, and this may reflect my chemical naïveté, is that the authors bury one of their more exciting implications until the final paragraph. The idea that partitioning into the aerosol phase may extend the effective atmospheric lifetime by shielding these compounds from gas-phase oxidation is very exciting and deserves further exploration. It certainly suggests some behavioural studies where insect detection of aerosols is explicitly examined. As a reader, I wish this idea had been presented as a possibility in the Introduction so that it could help frame the rest of the manuscript.

I realize the authors would probably prefer a finer-grained review with detailed criticisms, but as I said, in the critical area for those, I am underqualified. As a Biologist, however, I think this manuscript makes a valuable conceptual addition to the literature. I would prefer to see it restructured somewhat around the broader more complex idea of <lifetime> that they introduce at the end; I think its appeal to the Biology audience would be more obvious and the resulting citations more numerous. I look forward to citing Langford et al. when it appears.

Reviewer #2 (Remarks to the Author):

The manuscript entitled “The Differential Impact of Air Pollution on Insect Chemical Communication” by Langford et al. investigates the effects of air pollution on insect chemical signaling using a modeling framework grounded in gas-to-particle partitioning and atmospheric oxidation processes. The study adopts an interdisciplinary perspective that connects atmospheric chemistry with ecological function and fits well within the scope of Communications Earth & Environment. The methods are valid, the analysis is carefully executed, and the results provide useful insight into how pollution may influence pheromone lifetimes. The manuscript is clearly written and accessible to a broad audience. However, several aspects of the presentation and interpretation would benefit from clarification and restructuring to improve clarity and address some conceptual caveats prior to final acceptance.

Major comments:

1. Role and Usefulness of VBS Space and C*
With the exception of Figure 1, the repeated use of VBS space throughout the manuscript appears largely redundant and, at times, conceptually confusing. Because neither chemical oxidation nor physical partitioning alters the intrinsic C* of the parent molecules, the use of VBS space in Figures 2 and 3 is not clearly justified. These figures primarily illustrate temperature-driven changes in C*, which are already effectively conveyed in Figure 1. As presented, the VBS framework does not add new physical insight in these cases.
I encourage the authors to reconsider the figure design and to show more directly the impact of pollution on pheromone lifetime (Figure 2) and aerosol loss (Figure 3). This comment also applies to Figures S1–S5.
As a suggestion, Figure 1 could include multiple temperatures (e.g., three or four) in a single panel, eliminating the need for a supplementary figure (Figure S1, referenced on line 339 but currently missing). In addition, Figures 2, 3, and 4 could potentially be consolidated (see comment 4 below), which would streamline the narrative and reduce redundancy.

2. Quantification of Uncertainty and Sensitivity
The study relies on a limited subset of pheromones and is based largely on theoretical rate constants and model-derived parameters, which are inherently uncertain—an important point acknowledged by the authors in the Discussion. To further strengthen the conclusions, the manuscript would benefit from a more quantitative treatment of these uncertainties.
Specifically, the authors are encouraged to:
(1) compare rate constants derived from Episuite and GECKO-A with available laboratory measurements, where such data exist, in order to estimate uncertainties; and
(2) perform a sensitivity analysis illustrating how uncertainty bounds in these rate constants propagate into pheromone lifetime estimates.
Even a limited analysis for a small number of representative compounds would greatly enhance confidence in the robustness and generality of the results.

3. Impact of Yield Variability on C*
How much overestimation in C* is expected due to the variability of y0-activity coefficient (Line 180). Since in this study, only

C* of larger molecules are impacted by temperature- it might be worth noting the impact of y0.

4. Reconstructing Figure 4
A different representation of Figure 4 is recommended. In its current form, the figure is difficult to interpret without extensive cross-referencing to the text, and the primary takeaway is not immediately clear. It appears that the goal is not to contrast pollution levels per se, but rather to contextualize the relative contributions of chemical and physical loss to total lifetime reduction.

One possible approach would be to separate the figure into two panels: one representing pristine conditions and one representing polluted conditions. Including lifetime loss due to oxidation, aerosol partitioning and total as 3 separate bars, along with a secondary (right-hand) axis showing the original lifetimes, would provide helpful context and scale. In such a format, a revised Figure 4 could effectively integrate the information currently distributed across Figures 2, 3, and 4, clearly illustrating total lifetime loss and its chemical and physical components.

5. Discussion and conclusions
The statements in lines 532–539 of the Discussion are not directly supported by the results presented in the manuscript and are not substantiated by the cited literature. If these ideas are intended as broader speculation, this should be made explicit, and the text could either be removed or moved to the Introduction. As written, these statements also appear to conflict with the authors' conclusions beginning on line 543.

Minor comments:

1. Line 36, delete '}'
2. Line 150, superscript 10-4 to be corrected
3. Eq. (4), Please check whether C* should be capitalized
4. Lines 198- 215, Please standardize the notation and font for M – lines 198, 204 and 213 have different names. I suggest introducing it as particle OA mass already at the first occurrence.
5. Line 214, References for the chosen OA masses are needed, including where to expect such concentrations.
6. Figure S1, mentioned on line 339 is missing. See major comment # 1.
7. Line 392-394, please clarify the statement as to what is being compared.
8. Table 1, in the header line after Pristine, delete '('
9. Fig 3 (A), ylabel is different than the rest of the subplots.
10. Fig 4 caption is missing 30 oC.

Reviewer #3 (Remarks to the Author):

In COMMSENV-25-5566, the authors computationally examine the interactive effects of temperature, volatility, and oxidation on the lifetime of insect chemical signal compounds, focusing on well-studied pheromones and alarm compounds. I am very enthusiastic about the manuscript and believe it provides important context for the study of these compound types and their atmospheric processing. I only have a few suggestions/comments.

1. The authors present the effects of different oxidants on the the saturation concentration for a compounds under different environmental conditions. By first principles, I think this is an excellent starting point. However, I would urge the authors to expand their analyses to *when* the compounds are typically released, and how they change through time.

-Regarding the first point: for example, the sex pheromone compounds are released at dusk/night conditions (e.g., bombykal, bombykol). If the authors separate out the compounds based on their typical release times, how would this impact which are the dominant oxidants potentially controlling their concentrations and lifetimes?

-Second, I'm curious if the authors could place their *C calculations into a dynamic natural framing given the changes in temperature and oxidant concentrations over time; for example, using timeseries data during a few days time period or over a filtered time period of weeks to month to estimate sex pheromone and/or alarm pheromone *C. Perhaps using PvapT(t) via Clausius-Clapeyron or analogous. This would allow the authors to apply their calculations to dynamic historical timeseries conditions when a given locale was heavily polluted or pristine.

2. Lines 381-387: The compounds the authors refer to are not only pheromones, but also common plant and flower signal compounds that are emitted during both night and day. I would edit (or remove) these lines for clarity. I would suggest removing lines 388-391 since these compounds are used in many different ecological functions (not only as kairomones).

3. Lines 515-518: Please include references.

4. Lines 527-545: I am enthusiastic about the processes described here. The sinks and sources of these compounds can be dynamic. In sex pheromone trapping work, subsequent release from neighboring vegetation is one such example of potential reservoirs.

** Visit Nature Portfolio's author and referees' website at www.nature.com/authors for information about policies, services and author benefits**

Communications Earth & Environment is committed to improving transparency in authorship. As part of our efforts in this direction, we are now requesting that all authors identified as 'corresponding author' create and link their Open Researcher and Contributor Identifier (ORCID) with their account on the Manuscript Tracking System prior to acceptance. ORCID helps the scientific community achieve unambiguous attribution of all scholarly contributions. You can create and link your ORCID from the home page of the Manuscript Tracking System by clicking on 'Modify my Springer Nature account' and following the instructions in the link below. Please also inform all co-authors that they can add their ORCIDs to their accounts and that they must do so prior to acceptance.
https://www.springernature.com/gp/researchers/orcid/orcid-for-nature-research

For more information please visit http://www.springernature.com/orcid

If you experience problems in linking your ORCID, please contact the Platform Support Helpdesk.

Version 1:

Decision Letter:

** Please ensure you delete the link to your author home page in this e-mail if you wish to forward it to your coauthors **

Dear Dr Langford,

Your manuscript titled "The Differential Impact of Air Pollution on Insect Chemical Communication" has now been seen by our reviewers, whose comments appear below. In light of their advice we are delighted to say that we are happy, in principle, to publish a suitably revised version in Communications Earth & Environment.

We therefore invite you to revise your paper one last time to edit your manuscript to comply with our format requirements and to maximise the accessibility and therefore the impact of your work.

EDITORIAL REQUESTS:

Please review our specific editorial comments and requests regarding your manuscript in the attached "Editorial Requests Table".

*****Please take care to match our formatting and policy requirements. We will check revised manuscript and return manuscripts that do not comply. Such requests will lead to delays. *****

Please outline your response to each request in the right hand column. Please upload the completed table with your manuscript files as a Related Manuscript file.

If you have any questions or concerns about any of our requests, please do not hesitate to contact me.

SUBMISSION INFORMATION:

In order to accept your paper, we require the files listed at the end of the Editorial Requests Table; the list of required files is also available at https://www.nature.com/documents/commsj-file-checklist.pdf .

OPEN ACCESS:

Communications Earth & Environment is a fully open access journal. Articles are made freely accessible on publication. For further information about article processing charges, open access funding, and advice and support from Nature Portfolio, please visit https://www.nature.com/commsenv/open-access

At acceptance, you will be provided with instructions for completing the open access licence agreement on behalf of all authors. This grants us the necessary permissions to publish your paper. Additionally, you will be asked to declare that all required third party permissions have been obtained, and to provide billing information in order to pay the article-processing charge (APC).

Please use the following link to submit the above items:
Link Redacted

** This url links to your confidential home page and associated information about manuscripts you may have submitted or be reviewing for us. If you wish to forward this email to co-authors, please delete the link to your homepage first **

We hope to hear from you within two weeks; please let us know if you need more time.

Best regards,

Alice Drinkwater, PhD
Associate Editor
Communications Earth & Environment
Consulting Editor
Communications Sustainability

REVIEWERS' COMMENTS:

Reviewer #2 (Remarks to the Author):

The manuscript presents a novel and interdisciplinary investigation of the effects of air pollution on insect chemical signaling, employing a modeling framework based on gas–particle partitioning and atmospheric oxidation processes. During this round of revisions, the authors have done an excellent job addressing the reviewer comments in a thorough, point-by-point manner. All concerns have been carefully considered and satisfactorily resolved. The revisions have notably improved the clarity and quality of the manuscript, particularly through enhanced text and figure captions that are now more accessible to readers from multiple fields. Furthermore, the inclusion of sensitivity analyses, improvements in readability, and the reconstruction of figure 4 have strengthened the overall presentation. In its revised form, the manuscript represents a valuable contribution to both fields of atmospheric chemistry and ecology and is fit to be published in Communications Earth & Environment.

Reviewer #3 (Remarks to the Author):

The authors have addressed my limited comments. I am very enthusiastic about the manuscript and associated research work.

** Visit Nature Portfolio's author and referees' website at www.nature.com/authors for information about policies, services and author benefits**

Open Access This Peer Review File is licensed under a Creative Commons Attribution 4.0 International License, which permits use, sharing, adaptation, distribution and reproduction in any medium or format, as long as you give appropriate credit to the original author(s) and the source, provide a link to the Creative Commons license, and indicate if changes were made.
In cases where reviewers are anonymous, credit should be given to 'Anonymous Referee' and the source.
The images or other third party material in this Peer Review File are included in the article's Creative Commons license, unless indicated otherwise in a credit line to the material. If material is not included in the article's Creative Commons license and your intended use is not permitted by statutory regulation or exceeds the permitted use, you will need to obtain permission directly from the copyright holder.
To view a copy of this license, visit https://creativecommons.org/licenses/by/4.0/

We thank the three reviewers for their positive comments on our manuscripts and helpful suggestions for its improvement. Below we address each in turn.

Reviewer 1.

RC: My impression, and this may reflect my chemical naïveté, is that the authors bury one of their more exciting implications until the final paragraph. The idea that partitioning into the aerosol phase may extend the effective atmospheric lifetime by shielding these compounds from gas-phase oxidation is very exciting and deserves further exploration. It certainly suggests some behavioural studies where insect detection of aerosols is explicitly examined. As a reader, I wish this idea had been presented as a possibility in the Introduction so that it could help frame the rest of the manuscript.

AR: The idea that partitioning of gas phase compounds to aerosol shields them from gas-phase oxidation is not new and has been demonstrated for pesticides in the following paper: Socorro J, Durand A, Temime-Roussel B, Gligorovski S, Wortham H, Quivet E. The persistence of pesticides in atmospheric particulate phase: An emerging air quality issue. Sci Rep. 2016 Sep 15;6:33456. doi: 10.1038/srep33456. PMID: 27628441; PMCID: PMC5024296.

We have included this reference in the revised manuscript. The reviewer is correct that this suggests behavioural studies where insect detection of aerosols is explicitly examined and indeed this work has already been undertaken by one of the authors of this manuscript see reference 35: M. Jaffar-Bandjee, F. Figon, P. Clémençon, J.-B. Renard, J. Casas, Aerosol Alteration of Behavioral Response to Pheromone in Bombyx mori. *Journal of Chemical Ecology* 49, 353-362 (2023).

Reviewer 2

RC: 1. Role and Usefulness of VBS Space and C*
With the exception of Figure 1, the repeated use of VBS space throughout the manuscript appears largely redundant and, at times, conceptually confusing. Because neither chemical oxidation nor physical partitioning alters the intrinsic C* of the parent molecules, the use of VBS space in Figures 2 and 3 is not clearly justified. These figures primarily illustrate temperature-driven changes in C*, which are already effectively conveyed in Figure 1. As presented, the VBS framework does not add new physical insight in these cases.
I encourage the authors to reconsider the figure design and to show more directly the impact of pollution on pheromone lifetime (Figure 2) and aerosol loss (Figure 3). This comment also applies to Figures S1–S5.

AR: We thank the reviewer for raising this point and agree that the intrinsic *C** of a parent compound is not altered by either oxidation or partitioning. Our intention in Figure 2 was not to suggest otherwise, but rather to use *C** as an organising coordinate to visualise why the two pheromone classes diverge in atmospheric lifetime under different pollution conditions.

Plotting lifetime against *C** reveals, in a single 2-D space, the two mechanistic drivers of lifetime differences across the pheromone classes. The first is volatility (captured by *C**), which determines how much of the compound remains in the gas phase and is therefore available to react. The second is the intrinsic chemical reactivity of the molecule, which is

higher for the larger, more unsaturated sex pheromones. These molecules typically possess multiple double bonds and extended aliphatic chains, providing more reactive sites for OH, O□ and NO□ attack and therefore faster gas-phase rate constants than the smaller, more saturated alarm pheromones. Thus, the horizontal separation in *C** space also indirectly reflects structural trends that give rise to different oxidative loss rates.

Removing the *C** axis and showing lifetime only would obscure this mechanistic separation and collapse the comparison into a single-dimension plot, in which the physical and chemical reasons for the divergence in lifetime between alarm and sex pheromones become much less interpretable. We therefore believe that retaining *C** on the x-axis is essential for communicating the physical basis of the observed differences.

We will clarify the caption to explicitly state that *C** is an invariant property of the parent molecule (at fixed temperature and γ0) and is used here solely as a volatility coordinate, not as a quantity altered by the processes being examined, and that structural differences between the compound classes also contribute to their differing gas-phase reactivities.

"**Figure 2.** ***Alarm and sex pheromones plotted in volatility basis set (VBS) space, using C* as an invariant volatility coordinate (defined at fixed temperature and γ□). Atmospheric lifetime is shown on the y-axis. Open circles indicate lifetimes under pristine atmospheric conditions (Table 1), and arrows show the reduction in lifetime under highly polluted conditions. Panel A represents a typical daytime scenario (25 °C) with daytime oxidant levels; Panel B represents a nocturnal scenario (15 °C) with night-time oxidant levels. The C* axis is used here to organise compounds along the volatility continuum and to illustrate how volatility and intrinsic chemical reactivity together drive the divergence in atmospheric lifetimes between alarm and sex pheromones***."

For Figure 3, however, the use of C* is not optional but is mathematically required. The aerosol-phase fraction is defined by the standard absorptive-partitioning expression:

$F = \frac{1}{1+\frac{C^*}{M}}$. which depends explicitly and solely on the ratio C^*/M. Because *C** directly controls where each compound sits on the volatility continuum, and because M (organic aerosol mass) represents the pollution burden, Figure 3 necessarily embeds *C** as a central coordinate. Without *C**, the partitioning line and the differing responses of the compound classes cannot be derived or meaningfully shown.

In other words, while Figure 2 uses *C** as an organising axis for interpretability, Figure 3 depends on *C** mechanistically: the divergence in aerosol loss between compound classes arises *because* of their contrasting *C** values. Removing or replacing *C** would obscure the functional dependence of F on volatility and would disconnect the figure from the underlying partitioning physics.

For clarity, we will strengthen the caption and text to emphasise that (i) *C** is an intrinsic thermodynamic property, and (ii) its inclusion here reflects the structure of the partitioning expression rather than an assumption that *C** is modified by atmospheric processes. Beyond this clarification, we believe Figure 3 in its present form most faithfully represents the physical model being used.

"**Figure 3.** ***Alarm and sex pheromones plotted in volatility basis set (VBS) space, with the aerosol-phase fraction (F) on the y-axis. Panels show temperatures of 30 °C (A), 25 °C (B), 15 °C (C), and 5 °C (D), for aerosol mass loadings of 0.8, 25, and 50 μg m $^{-3}$.***

Because the aerosol-phase fraction is defined by F = 1 / (1 + C*/M), C* serves as the volatility coordinate that determines how each compound partitions between gas and particle phases under different pollution levels."

RC: Figure S1, referenced on line 339 but currently missing

AR: This has been added

RC: In addition, Figures 2, 3, and 4 could potentially be consolidated (see comment 4 below), which would streamline the narrative and reduce redundancy.

AR: For the reasons outlined above, we prefer to keep figures 1-3 in their current arrangement. We address figure 4 below.

RC: compare rate constants derived from Episuite and GECKO-A with available laboratory measurements, where such data exist, in order to estimate uncertainties and perform a sensitivity analysis illustrating how uncertainty bounds in these rate constants propagate into pheromone lifetime estimates. Even a limited analysis for a small number of representative compounds would greatly enhance confidence in the robustness and generality of the results.

AC: We thank the reviewer for this constructive suggestion. As indicated in Table S1, we already incorporate all known laboratory determinations directly into our analysis (shown in bold). However, as the table illustrates, such measurements are extremely limited and available almost exclusively for the alarm pheromones, which are small, volatile molecules for which gas-phase kinetics have been studied in the atmospheric chemistry literature.

For the sex pheromones, we found no published laboratory OH, O_3, or NO_3 rate constants, a situation consistent with both the chemical ecology and atmospheric chemistry literature. This absence reflects a long-standing experimental limitation: sex pheromones are large, very low-volatility, surface-active compounds, typically with vapour pressures below 10^{-6} atm at ambient temperature. Given the near-complete absence of laboratory data for sex pheromones, a formal uncertainty propagation based on measured–modelled differences can only be meaningfully performed for the alarm-pheromone subset. The revised SI now shows plots of pheromone lifetimes calculated with rate constants derived from SAR vs those available in the literature.

The manuscript text has been changed as follows:

"While these tools provide valuable first-order estimates, they carry inherent uncertainties (36). For example, a sensitivity analysis comparing lifetimes derived using structural activity relationships versus those based on literature values suggested lifetimes to be overestimated by an average of 27% (Fig. S8 of the SI)."

The SI has been updated with the following:

"S1.5 Use of rate constants derived from structural activity relationships – sensitivity study

To assess the reliability of structure–activity relationship (SAR) methods for estimating atmospheric lifetimes, we compared SAR-derived rate constants with those obtained from literature values. Lifetimes with respect to OH, O_3 and NO_3 were combined into a single analysis to evaluate performance across the full range of oxidants relevant to pheromone degradation.

Figure S8 shows SAR-derived lifetimes (y-axis) plotted against literature-based lifetimes (x-axis), with the intercept constrained to zero to reflect the expectation that a compound with zero lifetime in the

reference dataset must also have zero SAR-predicted lifetime. The resulting regression, y=1.2682 x, exhibits a strong linear relationship across the dataset (R^2 = 0.99). The slope indicates that SAR-based estimates predict lifetimes that are, on average, approximately 27% longer than those derived from literature rate constants, implying a corresponding underestimation of oxidant reaction rate constants by SAR methodologies.

However, despite this clear overall trend, the data show noticeable scatter. In particular, several of the more reactive (short-lived) compounds fall below the line of best fit, indicating instances where SAR methods overestimate their reactivity and therefore underestimate their atmospheric lifetimes. This behaviour is consistent with the known compound-specific uncertainties inherent in SAR formulations.

Overall, the combined analysis demonstrates that SAR-derived rate constants provide broadly reliable first-order estimates of atmospheric lifetimes across structurally diverse pheromones and oxidants, while also highlighting the expected variability for individual compounds."

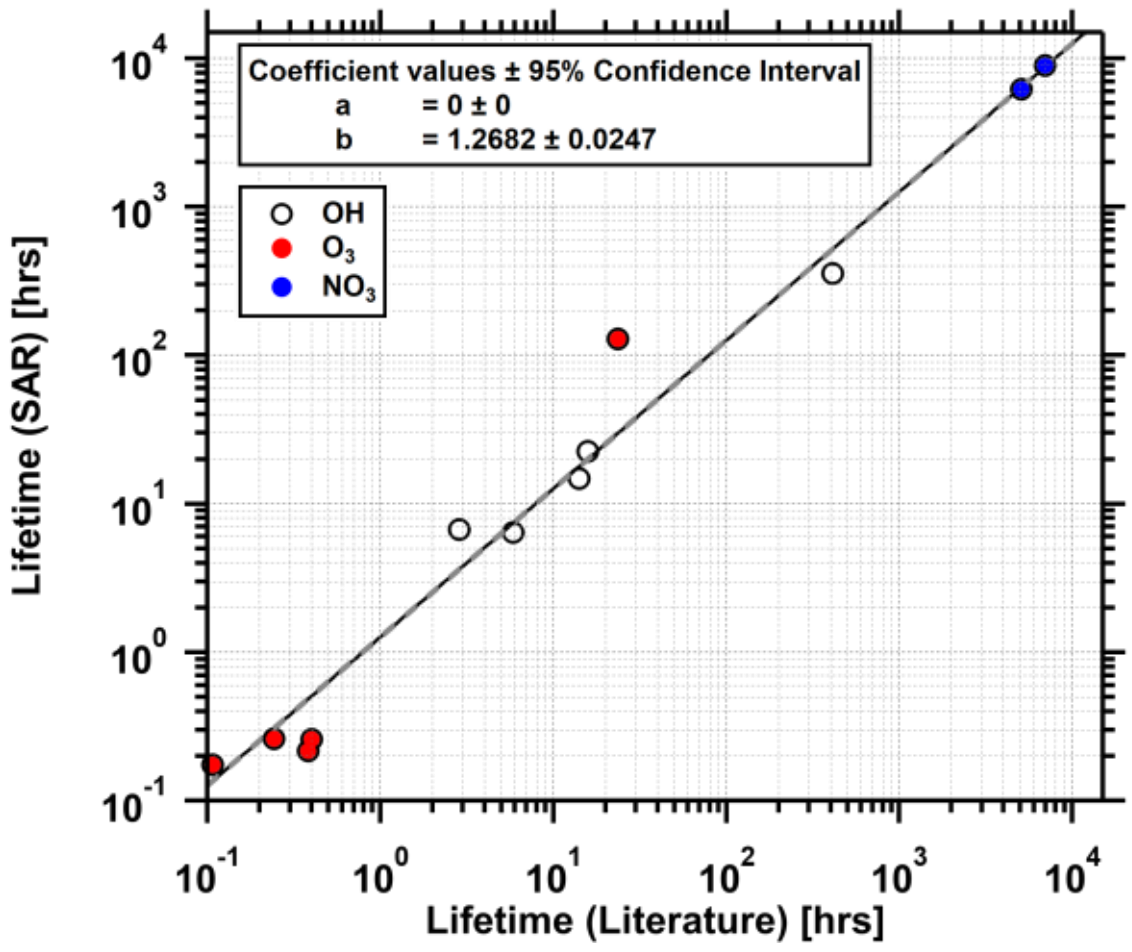

Figure S8. Comparison of SAR-derived atmospheric lifetimes with literature-based lifetimes for OH, O_3 and NO_3 oxidation."

RC: How much overestimation in C* is expected due to the variability of y0-activity coefficient (Line 180). Since in this study, only C* of larger molecules are impacted by temperature- it might be worth noting the impact of y0.

AR: We thank the reviewer for raising this important point. As the reviewer notes, C* scales linearly with the condensed-phase activity coefficient γ□, and therefore uncertainties in γ□ can directly influence the magnitude of C^*, particularly for the larger, low-volatility molecules that dominate the sex-pheromone class. To address this, we have now included a dedicated γ□ sensitivity analysis in the Supplementary Information. In this analysis, we explored representative γ□ ranges for the different functional groups present in our dataset and quantified how these propagate into revised C^* values and the resulting aerosol-phase fractions F at both 15 °C and 25 °C across the three OA loadings used in the study.

The results show that C* increases in proportion to γ□, leading to reduced aerosol partitioning under high-γ□ scenarios. However, these shifts do not alter our qualitative conclusions. Gas-phase-dominated alarm pheromones (with large C^* relative to aerosol mass) remain essentially unaffected, while sex-pheromone-like LVOC/SVOCs—already close to the $C^*/M \sim 1$ boundary—show predictable compression of F under elevated γ□. We now reference this sensitivity study in the main text.

"For simplicity, we set y0 = 1 for all molecules, recognising that this may overestimate C for larger, less-ideal species; a sensitivity analysis exploring the influence of y0 is provided in the Supplementary Information."*

The following sensitivity study has been added to the SI:

In the main manuscript, for simplicity, Equation 2 sets the condensed-phase activity coefficient γ_0 to 1, effectively assuming ideal mixing within the organic aerosol phase. In reality, γ_0 can deviate substantially from unity, particularly for large, low-volatility, multifunctional molecules. Smaller volatile organics generally mix nearly ideally and therefore exhibit $\gamma_0 \approx 1$. In contrast, long-chain insect sex pheromones (approximately C_{12}–C_{20}) tend to behave much less ideally within organic aerosol. Their extended hydrocarbon backbones, low polarity and limited solubility can lead to elevated activity coefficients, with plausible γ_0 values in the range of 3–10. The upper end of this range is most relevant for the very low-volatility C_{18}–C_{20} pheromones, whereas mid-chain C_{12}–C_{16} species likely occupy the lower portion of this interval. Because C scales linearly with γ_0, increases in γ_0 have the strongest influence on the lowest-volatility pheromones, for which even modest fractional changes in C* can meaningfully alter the predicted gas–particle partitioning. Table Sx illustrates this effect by presenting C* and partitioning fractions F across a range of γ_0 values (1, 3, 5, 10) for representative low-volatility sex pheromones under several aerosol-loading scenarios at 15 °C.*

Table S4. Sensitivity of aerosol-phase fraction *F* to condensed-phase non-ideality (γ_0) at 15 °C. Saturation concentrations *C (µg m⁻³) and resulting aerosol-phase fractions *F* (%) are shown for each compound under three organic-aerosol loadings: Pristine (0.8 µg m⁻³), Polluted (25 µg m⁻³), and Highly Polluted (50 µg m⁻³). *F* is calculated at 15 °C using $F=1/(1+C^*/M)$, with *C** scaled by the activity coefficient γ_0 (1, 3, 5, 10). Larger γ_0 values increase *C** and reduce *F*, illustrating the influence of non-ideality on partitioning for low-volatility pheromones.**

Compound	Scenario	C* [µg m^{-3}]	F (γ_0 = 1) [%]	F (γ_0 = 3) [%]	F (γ_0 = 5) [%]	F (γ_0 = 10) [%]
(Z)-11-Hexadecen-1-ol	Pristine (0.8 µg m^{-3})	8.05	0.09	0.03	0.02	0.01
	Polluted (25 µg m^{-3})	8.05	0.76	0.51	0.38	0.24
	Highly Polluted (50 µg m^{-3})	8.05	0.86	0.67	0.55	0.38
(10Z,12E)-Hexadeca-10,12-dien-1-OL (Bombykol)	Pristine (0.8 µg m^{-3})	10.39	0.07	0.03	0.02	0.01
	Polluted (25 µg m^{-3})	10.39	0.71	0.45	0.32	0.19
	Highly Polluted (50 µg m^{-3})	10.39	0.83	0.62	0.49	0.32
(E,E)-10,12-hexadecadien-1-ol	Pristine (0.8 µg m^{-3})	10.39	0.07	0.03	0.02	0.01
	Polluted (25 µg m^{-3})	10.39	0.71	0.45	0.32	0.19
	Highly Polluted (50 µg m^{-3})	10.39	0.83	0.62	0.49	0.32
	Pristine (0.8 µg m^{-3})	21.37	0.04	0.01	0.01	0.00

(Z)-11-Octadecenyl acetate	Polluted (25 μg m^{-3})	21.37	0.54	0.28	0.19	0.10
	Highly Polluted (50 μg m^{-3})	21.37	0.70	0.44	0.32	0.19
(Z)-11-Octadecen-1-ol	Pristine (0.8 μg m^{-3})	27.58	0.03	0.01	0.01	0.00
	Polluted (25 μg m^{-3})	27.58	0.48	0.23	0.15	0.08
	Highly Polluted (50 μg m^{-3})	27.58	0.64	0.38	0.27	0.15
(13Z)-13-Hexadecen-11-yn-1-yl acetate	Pristine (0.8 μg m^{-3})	32.09	0.02	0.01	0.00	0.00
	Polluted (25 μg m^{-3})	32.09	0.44	0.21	0.13	0.07
	Highly Polluted (50 μg m^{-3})	32.09	0.61	0.34	0.24	0.13

4. Reconstructing Figure 4

A different representation of Figure 4 is recommended. In its current form, the figure is difficult to interpret without extensive cross-referencing to the text, and the primary takeaway is not immediately clear. It appears that the goal is not to contrast pollution levels per se, but rather to contextualize the relative contributions of chemical and physical loss to total lifetime reduction.

One possible approach would be to separate the figure into two panels: one representing pristine conditions and one representing polluted conditions. Including lifetime loss due to oxidation, aerosol partitioning and total as 3 separate bars, along with a secondary (right-hand) axis showing the original lifetimes, would provide helpful context and scale. In such a format, a revised Figure 4 could effectively integrate the information currently distributed across Figures 2, 3, and 4, clearly illustrating total lifetime loss and its chemical and physical components.

AR: We thank the reviewer for the constructive suggestion. Figure two is designed to illustrate the magnitude of lifetime reduction in response to air pollution, capturing both gas phase oxidation and aerosol partitioning. By contrast, figure four is designed specifically to highlight what fraction is due to partitioning to aerosol. We have tried combing both into a single figure but found this not to work due to the need for a log scale for the lifetime, which then masks the relative contributions. Following the reviewer's advice, we have redesigned Figure 4 to include two panels (pristine and highly polluted aerosol loadings), each containing four subplots corresponding to 30 °C, 25 °C, 15 °C and 5 °C. Within each subplot, stacked bar charts now show the proportional contributions of gas-phase oxidation and aerosol partitioning to total lifetime reduction. We have reordered the pheromones Alarm->Alarm/Sex->Sex and used colours consistent with the earlier figures. We believe the revised figure is clearer, more intuitive, and better contextualises the role of aerosol-phase loss.

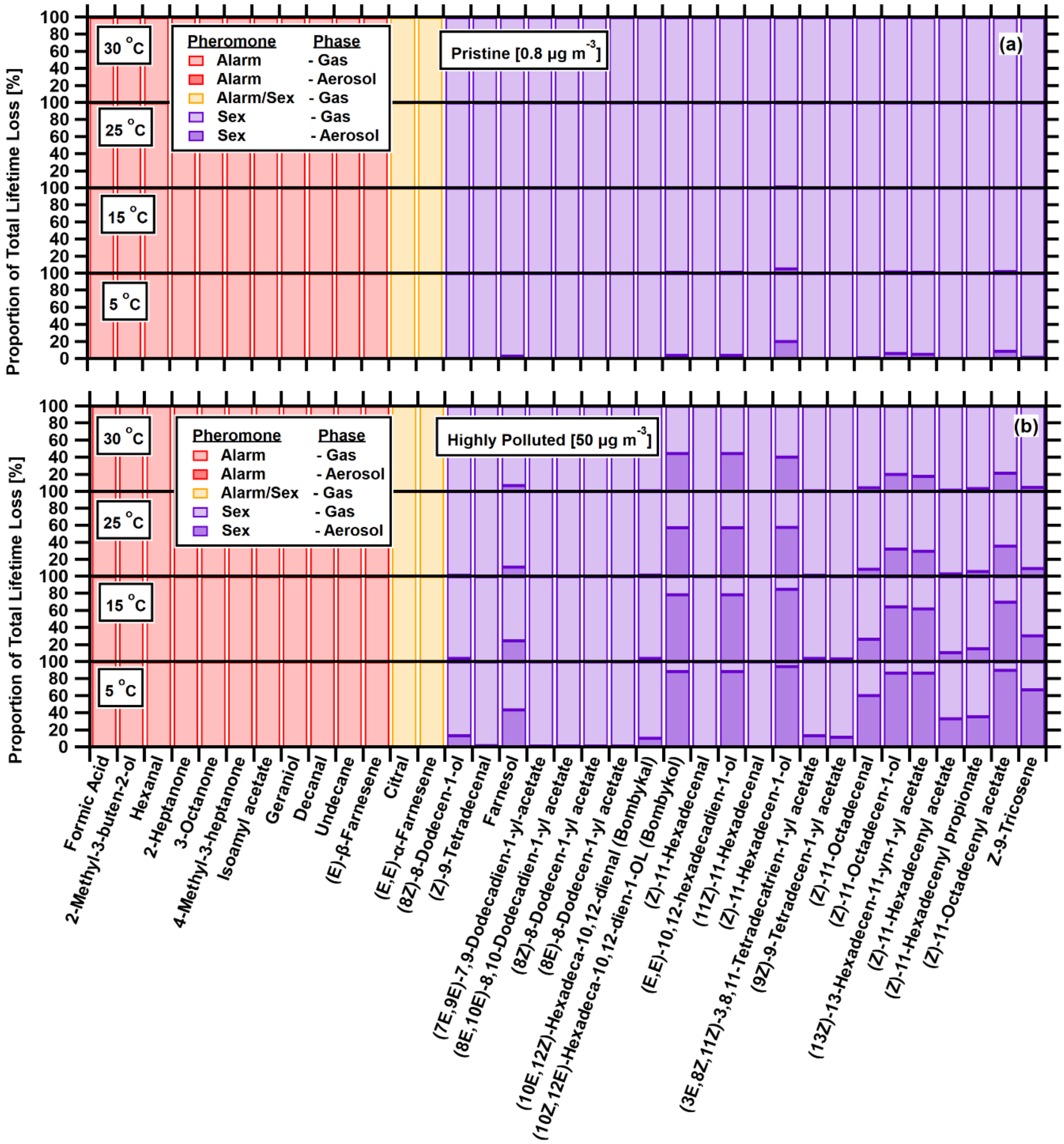

Figure 4. Proportional contributions of gas-phase oxidation and aerosol-phase partitioning to total pheromone lifetime loss under pristine and highly polluted conditions. Panel (a) represents pristine aerosol loadings (organic aerosol = 0.8 µg m⁻³) and panel (b) represents highly polluted conditions (organic aerosol = 50 µg m⁻³). Each panel contains four subplots corresponding to 30 °C, 25 °C, 15 °C, and 5 °C. The 30 °C and 25 °C conditions use day-time oxidant concentrations, while the 15 °C and 5 °C conditions use night-time oxidant concentrations, consistent with typical release timings of alarm and sex pheromones. Stacked bars show the percentage of total lifetime reduction attributable to gas-phase oxidation and aerosol-phase uptake under each environmental scenario.

RC: The statements in lines 532–539 of the Discussion are not directly supported by the

results presented in the manuscript and are not substantiated by the cited literature. If these ideas are intended as broader speculation, this should be made explicit, and the text could either be removed or moved to the Introduction. As written, these statements also appear to conflict with the authors' conclusions beginning on line 543.

AR: We thank the reviewer for this helpful comment. The sentence in question refers to the potential for the functional lifetime of pheromones to be extended once in the aerosol phase, as particle-bound compounds may experience reduced exposure to gas-phase oxidants. The reviewer is correct that this mechanism was not directly measured in our study. In the revised manuscript, we have therefore (i)added a citation to support plausibility—*Socorro et al.* (2016, *Scientific Reports*, 6, 33456), who demonstrate extended persistence of semi-volatile pesticides when particle-bound owing to slower oxidation and (ii) made the speculative nature explicit by framing the extension to pheromones as a speculative possibility by analogy. Finally, we note that there is no conflict with our conclusions beginning at line 543, which concern the functional lifetime of the gas-phase signal. In other words, even if condensed-phase residence were to increase chemical persistence, aerosol partitioning removes compounds from the biologically relevant gas phase, so the functional signal available for long-range communication is still reduced.

"This phase transition does not reduce atmospheric lifetime in the conventional chemical sense, but it does alter the physical state and potentially the bioavailability of the signal. For example, semi-volatile pesticides that partition to aerosols have been shown to exhibit extended lifetimes with respect to gas-phase oxidants (Socorro et al., 2016). By analogy, we note the speculative possibility that semi-volatile pheromones may gain a similar degree of protection once partitioned to aerosol, thereby preserving their chemical integrity and extending their detectable lifetime."

Minor comments:

1. Line 36, delete '}'

AR: Done

2. Line 150, superscript 10-4 to be corrected

AR: Done

3. Eq. (4), Please check whether C* should be capitalized

AR: Good catch – changed.

4. Lines 198- 215, Please standardize the notation and font for M – lines 198, 204 and 213 have different names. I suggest introducing it as particle OA mass already at the first occurrence.

AR: Thank you – this has been changed accordingly

5. Line 214, References for the chosen OA masses are needed, including where to expect such concentrations.

AR: These have been added *“Therefore, we consider only the organic fraction of the total aerosol and assume M = 0.8 μg m⁻³ for pristine conditions representative of the remote Amazon (26), M = 25 μg m⁻³ for moderately polluted conditions characteristic of rural locations downwind of urban areas (27), and M = 50 μg m⁻³ for highly polluted conditions typical of severely impacted urban regions (28).”*

26. S. T. Martin *et al.*, An overview of the Amazonian Aerosol Characterization Experiment 2008 (AMAZE-08). *Atmos. Chem. Phys.* **10**, 11415-11438 (2010).
27. Y. Zhang *et al.*, Aerosol chemistry and particle growth events at an urban downwind site in North China Plain. *Atmos. Chem. Phys.* **18**, 14637-14651 (2018).
28. J. M. Cash *et al.*, Seasonal analysis of submicron aerosol in Old Delhi using high-resolution aerosol mass spectrometry: chemical characterisation, source apportionment and new marker identification. *Atmos. Chem. Phys.* **21**, 10133-10158 (2021).

6. Figure S1, mentioned on line 339 is missing. See major comment # 1.

AR: This has been added

7. Line 392-394, please clarify the statement as to what is being compared.

AR: This has been changed to make it clear that it is the analysis shown in figures 2A and B: *“Repeating the analysis shown in figure 2 for temperatures more representative of equatorial climates (daytime 30 °C, nighttime 25 °C) showed negligible change in alarm pheromone lifetimes under pristine conditions, and a modest increase in sex pheromone lifetimes (+4.2%).”*

8. Table 1, in the header line after Pristine, delete ‘(‘

AR: Deleted

9. Fig 3 (A), ylabel is different than the rest of the subplots.

AR: Great catch – this has been changed to aerosol

10. Fig 4 caption is missing 30 oC.
AR: Figure 4 has been fully revised

Reviewer #3 (Remarks to the Author):

RC: In COMMSENV-25-5566, the authors computationally examine the interactive effects of temperature, volatility, and oxidation on the lifetime of insect chemical signal compounds, focusing on well-studied pheromones and alarm compounds. I am very enthusiastic about the manuscript and believe it provides important context for the study of these compound types and their atmospheric processing. I only have a few suggestions/comments.

1. The authors present the effects of different oxidants on the the saturation concentration for a compounds under different environmental conditions. By first principles, I think this is an excellent starting point. However, I would urge the authors to expand their analyses to *when* the compounds are typically released, and how they change through time.

-Regarding the first point: for example, the sex pheromone compounds are released at dusk/night conditions (e.g., bombykal, bombykol). If the authors separate out the compounds

based on their typical release times, how would this impact which are the dominant oxidants potentially controlling their concentrations and lifetimes?

AR: We thank the reviewer for this helpful observation. We note that this distinction is already incorporated into Figure 2, which explicitly contrasts daytime (a) and night-time (b) oxidant scenarios. As discussed in Section 2.5, alarm pheromones may be released at any time of day, but their oxidation is dominated by daytime oxidant levels. By contrast, most sex pheromones are typically released at dusk or during the night, when oxidant concentrations differ markedly from daytime values. These differences are reflected in the oxidant levels used in our calculations (summarised in Table 1).

Figure 2A presents an illustrative daytime scenario using representative daytime oxidant concentrations, while Figure 2B presents a night-time scenario using typical night-time oxidant levels. For clarity and comparability, both alarm and sex pheromones are shown in each panel; this is stated explicitly in the manuscript (lines 358–360). This allows the reader to directly assess how changes in oxidant regime influence lifetimes for both pheromone classes.

RC: Second, I'm curious if the authors could place their *C calculations into a dynamic natural framing given the changes in temperature and oxidant concentrations over time; for example, using timeseries data during a few days time period or over a filtered time period of weeks to month to estimate sex pheromone and/or alarm pheromone *C. Perhaps using PvapT(t) via Clausius-Clapeyron or analogous. This would allow the authors to apply their calculations to dynamic historical timeseries conditions when a given locale was heavily polluted or pristine.

AR: We thank the reviewer for this excellent suggestion. We fully agree that applying our framework to dynamic, time-resolved environmental conditions is a powerful next step. Indeed, we have already begun developing such an approach, and it will form the basis of a follow-up paper.

In that work, we plan to embed the present lifetime and volatility framework within an atmospheric chemistry transport model. This will allow us to calculate C^* dynamically using temperature-dependent vapour pressure (via Clausius–Clapeyron) and evolving oxidant fields, and thereby map realistic, time-varying atmospheric lifetimes of the pheromones across different regions and pollution regimes. Such a modelling framework will also enable us to examine historical conditions ranging from pristine to heavily polluted, as the reviewer suggests.

We believe this dynamic, model-integrated analysis is valuable but sits beyond the scope of the current paper, which is focused on establishing the foundational mechanisms and sensitivities. We are grateful to the reviewer for highlighting this direction, which we are actively pursuing.

2. Lines 381-387: The compounds the authors refer to are not only pheromones, but also common plant and flower signal compounds that are emitted during both night and day. I would edit (or remove) these lines for clarity. I would suggest removing lines 388-391 since these compounds are used in many different ecological functions (not only as kairomones).

AR: We thank the reviewer for this constructive comment. We agree that citral, geraniol, and the farnesenes are not exclusively insect pheromones, and that they are also emitted widely by plants as floral, vegetative, and stress-related volatiles. In the revised manuscript, we now explicitly acknowledge this broader biogenic context.

We have retained a brief discussion of these compounds because they stand out as clear outliers in our atmospheric lifetime analysis and therefore merit explanation.

"Within this group, however, several notable exceptions exhibit significantly shorter atmospheric lifetimes, including citral, geraniol, (E)-β-farnesene, and (E)-α-farnesene. Although these compounds are often discussed in the context of insect signalling—for example, citral and α-farnesene act as sex pheromones in some species (e.g., Pieris napi (30)) and as alarm signals in others (e.g., certain mites (31))—they are also widely emitted as plant volatiles. Likewise, geraniol and β-farnesene, recognised alarm pheromones in sycamore lace bugs (32) and aphids (33), respectively, are also common floral and vegetative emissions. Their short atmospheric lifetimes are therefore consistent with their high chemical reactivity as biogenic VOCs, rather than being uniquely tied to their roles in insect communication. Because these compounds participate in multiple ecological interactions—including, in some cases, functioning as kairomones—their rapid degradation may help restrict long-range detectability while still enabling effective short-range signalling."

3. Lines 515-518: Please include references.

AR: These have been added

For moths calling during daytime:

Levi-Zada, A., & Byers, J. A. (2021). Circadian rhythms of insect pheromone titer, calling, emission, and response: a review. *The Science of Nature*, *108*(5), 35.

For aerosol expelling :

Krasnoff, S. B., & Roelofs, W. L. (1988). Sex pheromone released as an aerosol by the moth Pyrrharctia isabella. *Nature*, *333*(6170), 263-265.

4. Lines 527-545: I am enthusiastic about the processes described here. The sinks and sources of these compounds can be dynamic. In sex pheromone trapping work, subsequent release from neighboring vegetation is one such example of potential reservoirs.

AR: Agreed. This very example is concept currently being explored by one of the authors.
