# Supplementary material for: The differential impact of air pollution on insect chemical communication
Source: Commun Earth Environ. 2026 May 16;7(1):631. doi: 10.1038/s43247-026-03608-7 (PMC13433313; doi:10.1038/s43247-026-03608-7)
Supplement: Supplementary file 2 [file 43247_2026_3608_MOESM2_ESM.pdf]

Supporting Information for

The differential impact of air pollution on insect chemical communication

Ben Langford[1*], Jonathan Williams[2,3] and Jerome Casas[4]

1. UK Centre for Ecology and Hydrology, Bush Estate, Penicuik, EH26 0QB, United Kingdom
2. Atmospheric Chemistry Department, Max Planck Institute for Chemistry, 55128, Mainz, Germany
3. Climate and Atmosphere Research Center, The Cyprus Institute, Nicosia, Cyprus.
4. Insect Biology Research Institute IRBI, University of Tours - CNRS, UMR 7261, Tours, France

*Ben Langford.

Email: benngf@ceh.ac.uk

This PDF file includes:

Supporting text

Figures S1 to S5

Tables S1 -S3

SI References

Supporting Notes

S1.1 Equatorial Temperatures

Figure S2 shows the same analysis presented in the main text using temperatures more representative of regions closer to the equator. Here a daytime temperature of 30 ^{o}C is used (Panel A) and a value of 25 ^{o}C is used at night (Panel B).

S1.2 Use of irreversible uptake model

Figures S3 to S6 show the fraction of each pheromone that would partition to the aerosol phase at temperatures of 5, 15, 25 and 30 ^{o}C, respectively, when the irreversible uptake model is used

(eq. 12) instead of the equilibrium equation employed in the main manuscript (e.g. eq. 5). While equation 5 assumes a rapid equilibrium, Eq. 12 is based on the principle that once partitioned to the aerosol, the pheromone is irreversibly bound and cannot re-volatilise. If this process holds, the losses of sex pheromones to the aerosol phase would be far larger than implied in the main manuscript.

S1.3 Mass accommodation coefficient – sensitivity study

The characteristic timescale for aerosol uptake (τ_p) is calculated using an estimate of the aerosol mass accommodation coefficient (α), which quantifies the probability that a gas-phase pheromone molecule will be absorbed into the organic aerosol upon collision. For highly reactive species, α may be enhanced by rapid reactions at the particle surface but could also be suppressed if the surface is already saturated. In the main manuscript, we assume $\alpha = 0.5$, reflecting moderately efficient uptake of reactive, semi-volatile organic compounds into organic aerosol. This value is consistent with literature estimates for reactive VOCs undergoing rapid uptake, particularly in systems where the aerosol phase is organic-rich and surface resistance is minimal.

While recent work has introduced more nuanced formulations of α (e.g., α_{eff}) that account for aerosol phase state, compound diffusivity, and particle-phase reactions (e.g., Shiraiwa & Pöschl, 2021[1]), our use of a fixed $\alpha = 0.5$ reflects a pragmatic choice given the limited physicochemical data available for pheromones and the simplified aerosol representation used here.

Figure S7 shows how τ_p changes in relation to the mass accommodation coefficient in a pristine atmosphere and under varying levels of pollution. Here we use the average τ_p value for the sex pheromones listed in Table S1, with the shaded areas showing ± σ.

Under highly polluted conditions, the characteristic timescale for aerosol uptake decreases from approximately 1 minute when α is set to 0.5, to just over 10 minutes when α is reduced to 0.05. To contextualise this, the gas-phase lifetime in the same nocturnal, polluted scenario averaged 2.7 hours, with the most reactive pheromones persisting for as little as 6 minutes. This comparison highlights that even under very inefficient partitioning ($\alpha = 0.05$), aerosol uptake still occurs at a rate comparable to or faster than gas-phase oxidation for most of the sex pheromones analysed.

S1.4 Condensed Phase Activity Coefficient, y0 – sensitivity study

In the main manuscript, for simplicity, Equation 3 sets the condensed-phase activity coefficient y_0 to 1, effectively assuming ideal mixing within the organic aerosol phase. In reality, y_0 can deviate substantially from unity, particularly for large, low-volatility, multifunctional molecules. Smaller volatile organics generally mix nearly ideally and therefore exhibit $y_0 \approx 1$. In contrast, long-chain insect sex pheromones (approximately C_{12}–C_{20}) tend to behave much less ideally within organic aerosol. Their extended hydrocarbon backbones, low polarity and limited solubility can lead to elevated activity coefficients, with plausible y_0 values in the range of 3–10. The upper end of this range is most relevant for the very low-volatility C_{18}–C_{20} pheromones, whereas mid-chain C_{12}–C_{16} species likely occupy the lower portion of this interval. Because C^* scales linearly with y_0, increases in y_0 have the strongest influence on the lowest-volatility pheromones, for which even modest fractional changes in C^* can meaningfully alter the predicted gas–particle partitioning. Table S4 illustrates this effect by presenting C^* and partitioning fractions F across a range of y_0 values (1, 3, 5, 10) for representative low-volatility sex pheromones under several aerosol-loading scenarios at 15 °C.

S1.5 Use of rate constants derived from structural activity relationships – sensitivity study

To assess the reliability of structure–activity relationship (SAR) methods for estimating atmospheric lifetimes, we compared SAR-derived rate constants with those obtained from literature values. Lifetimes with respect to OH, O_3 and NO_3 were combined into a single analysis to evaluate performance across the full range of oxidants relevant to pheromone degradation.

Figure S8 shows SAR-derived lifetimes (y-axis) plotted against literature-based lifetimes (x-axis), with the intercept constrained to zero to reflect the expectation that a compound with zero lifetime in the reference dataset must also have zero SAR-predicted lifetime. The resulting regression, $y=1.2682\,x$, exhibits a strong linear relationship across the dataset ($R^2 = 0.99$). The slope indicates that SAR-based estimates predict lifetimes that are, on average, approximately 27% longer than those derived from literature rate constants, implying a corresponding underestimation of oxidant reaction rate constants by SAR methodologies.

However, despite this clear overall trend, the data show noticeable scatter. In particular, several of the more reactive (short-lived) compounds fall below the line of best fit, indicating instances where SAR methods overestimate their reactivity and therefore underestimate their atmospheric lifetimes. This behaviour is consistent with the known compound-specific uncertainties inherent in SAR formulations.

Overall, the combined analysis demonstrates that SAR-derived rate constants provide broadly reliable first-order estimates of atmospheric lifetimes across structurally diverse pheromones and oxidants, while also highlighting the expected variability for individual compounds.

Supplementary Figures

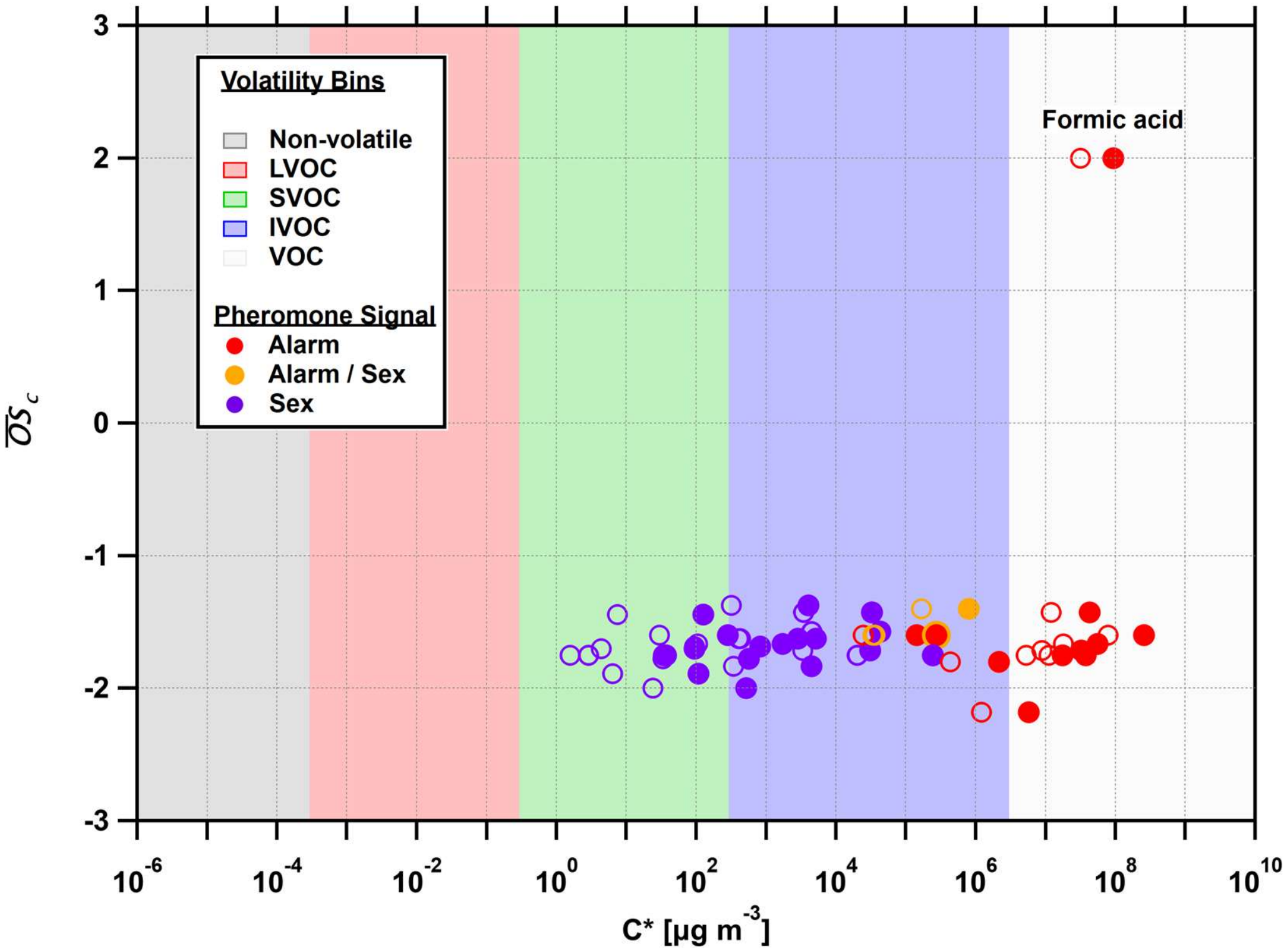

Figure S1 Common alarm and sex pheromones plotted in volatility basis set (VBS) space. Solid circles indicate data at 25 °C, while open circles represent data at 5 °C.

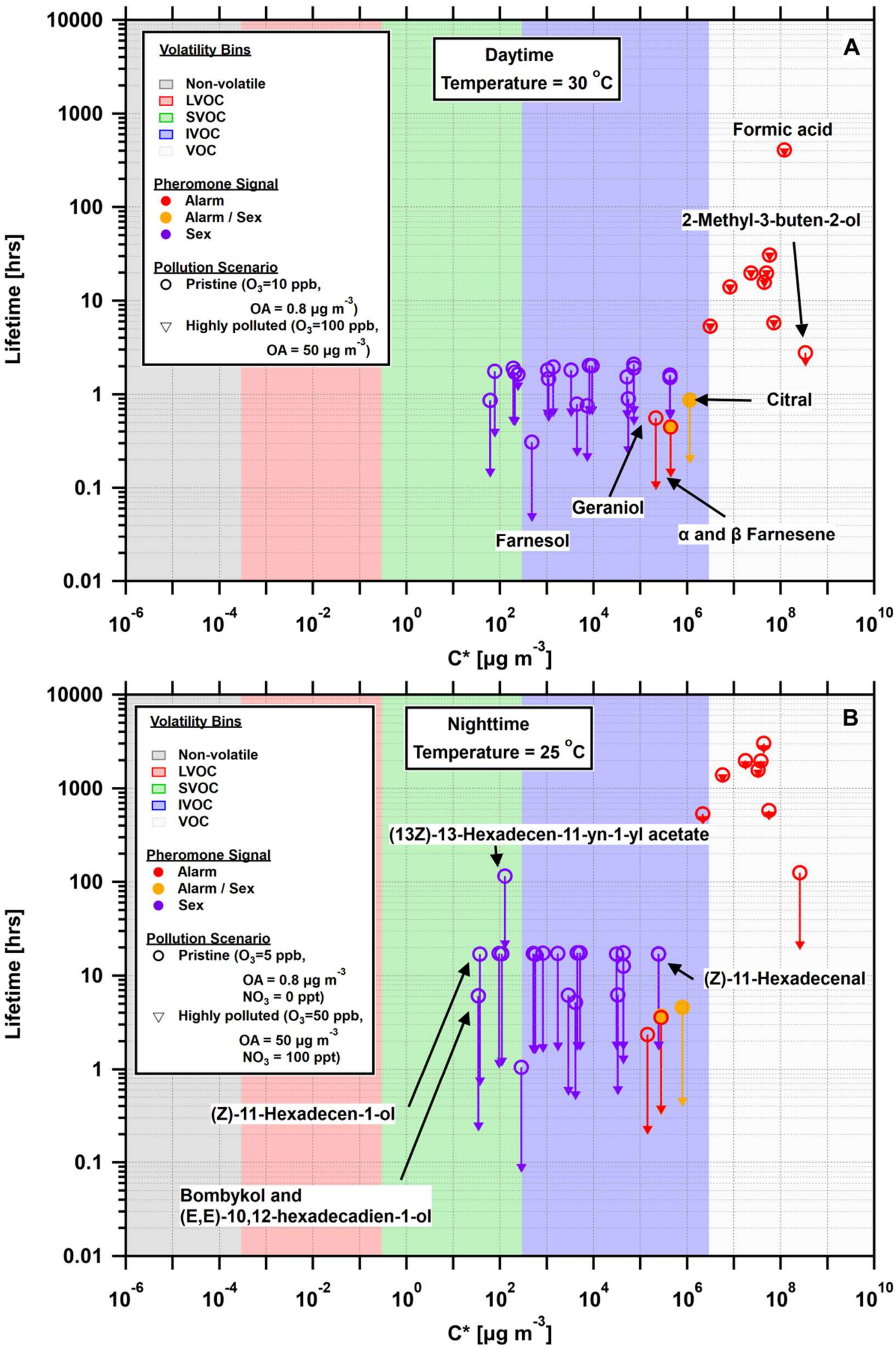

Figure S2. Alarm and sex pheromones mapped in volatility basis set (VBS) space, with atmospheric lifetime on the y-axis. Open circles indicate the position of each pheromone

under pristine atmospheric conditions (see Table 1), while arrows show the reduction in lifetime under highly polluted conditions. Panel A represents daytime oxidant conditions; Panel B represents a nocturnal scenario.

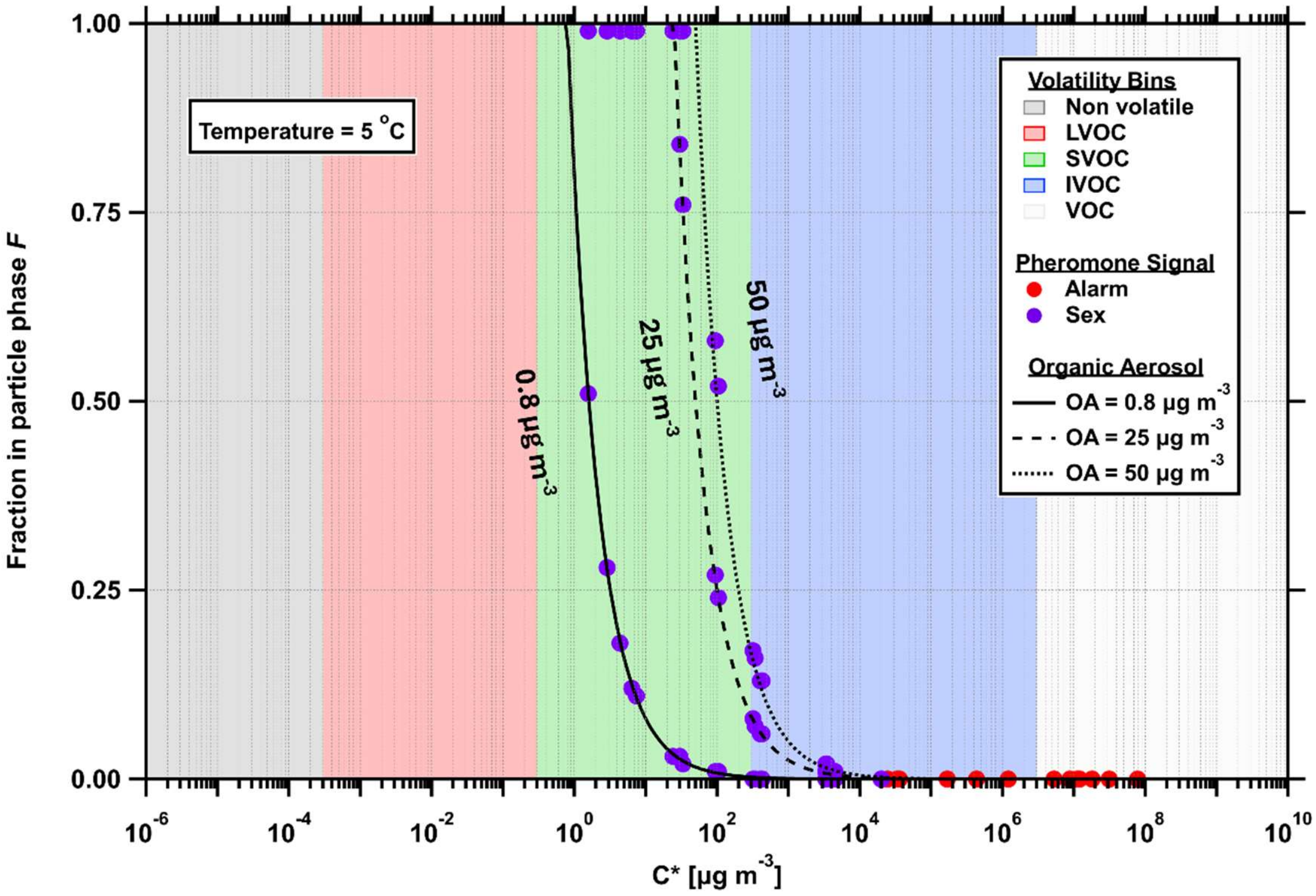

Figure S3 Common alarm and sex pheromones mapped in volatility basis set (VBS) space, with the fraction partitioned into the aerosol phase calculated using the irreversible uptake model (eq. 12) and shown on the y-axis. Results are presented for a temperature of 5 °C and for aerosol mass loadings of 0.8, 25, and 50 µg m^{-3}.

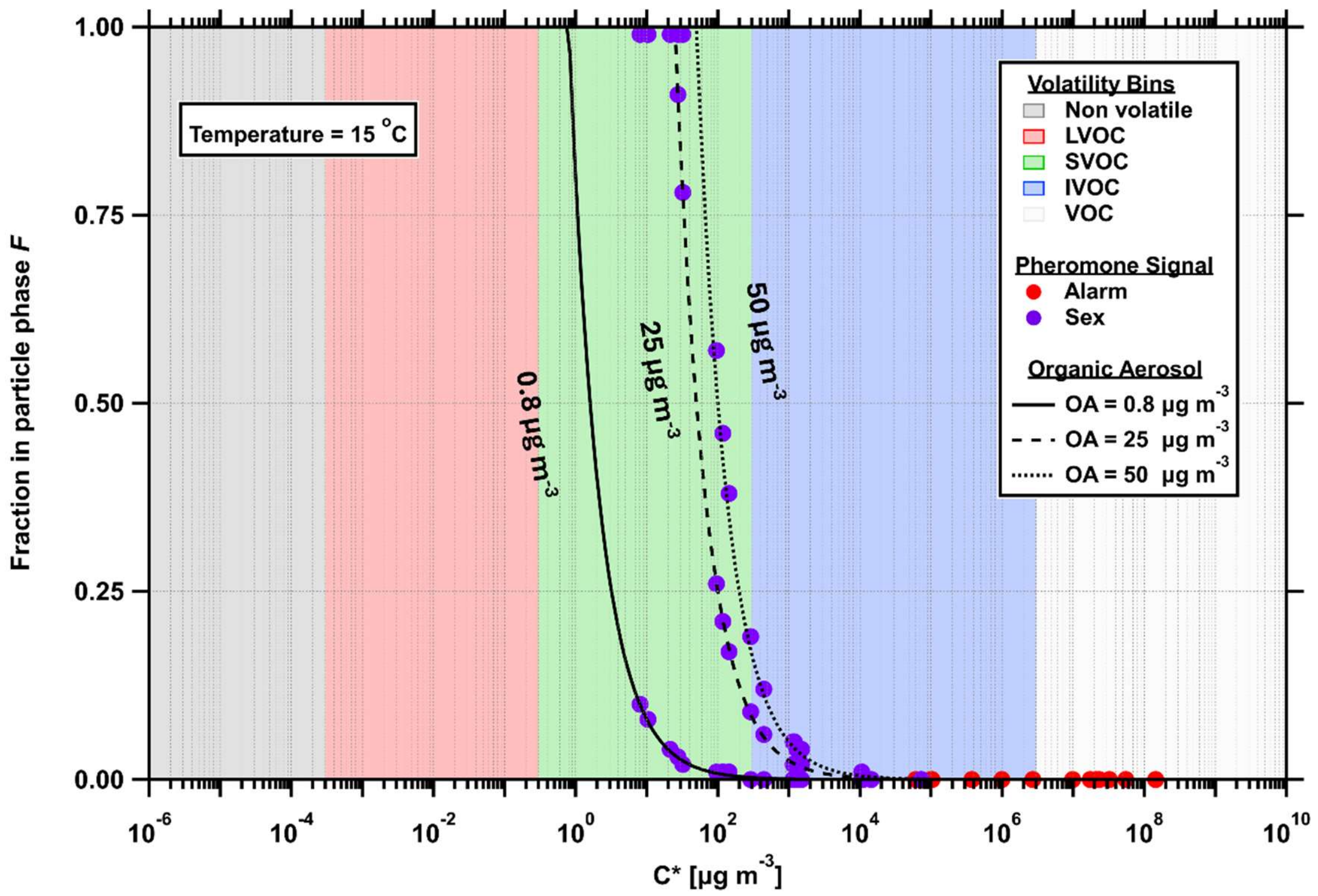

Figure S4 Common alarm and sex pheromones mapped in volatility basis set (VBS) space, with the fraction partitioned into the aerosol phase calculated using the irreversible uptake model (eq. 12) and shown on the y-axis. Results are presented for a temperature of 15 °C and for aerosol mass loadings of 0.8, 25, and 50 µg m^{-3}.

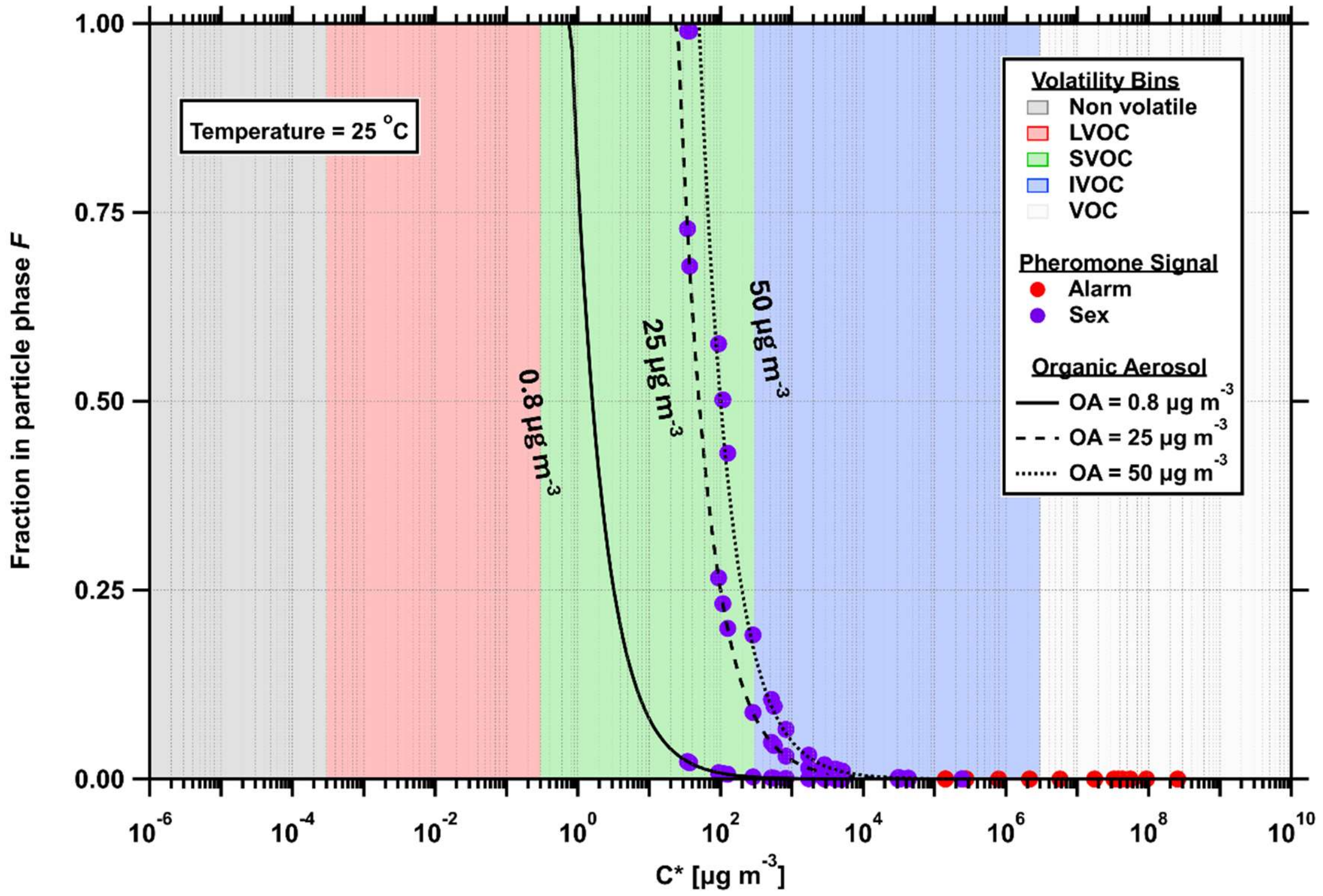

Figure S5 Common alarm and sex pheromones mapped in volatility basis set (VBS) space, with the fraction partitioned into the aerosol phase calculated using the irreversible uptake model (eq. 12) and shown on the y-axis. Results are presented for a temperature of 25 °C and for aerosol mass loadings of 0.8, 25, and 50 µg m^{-3}.

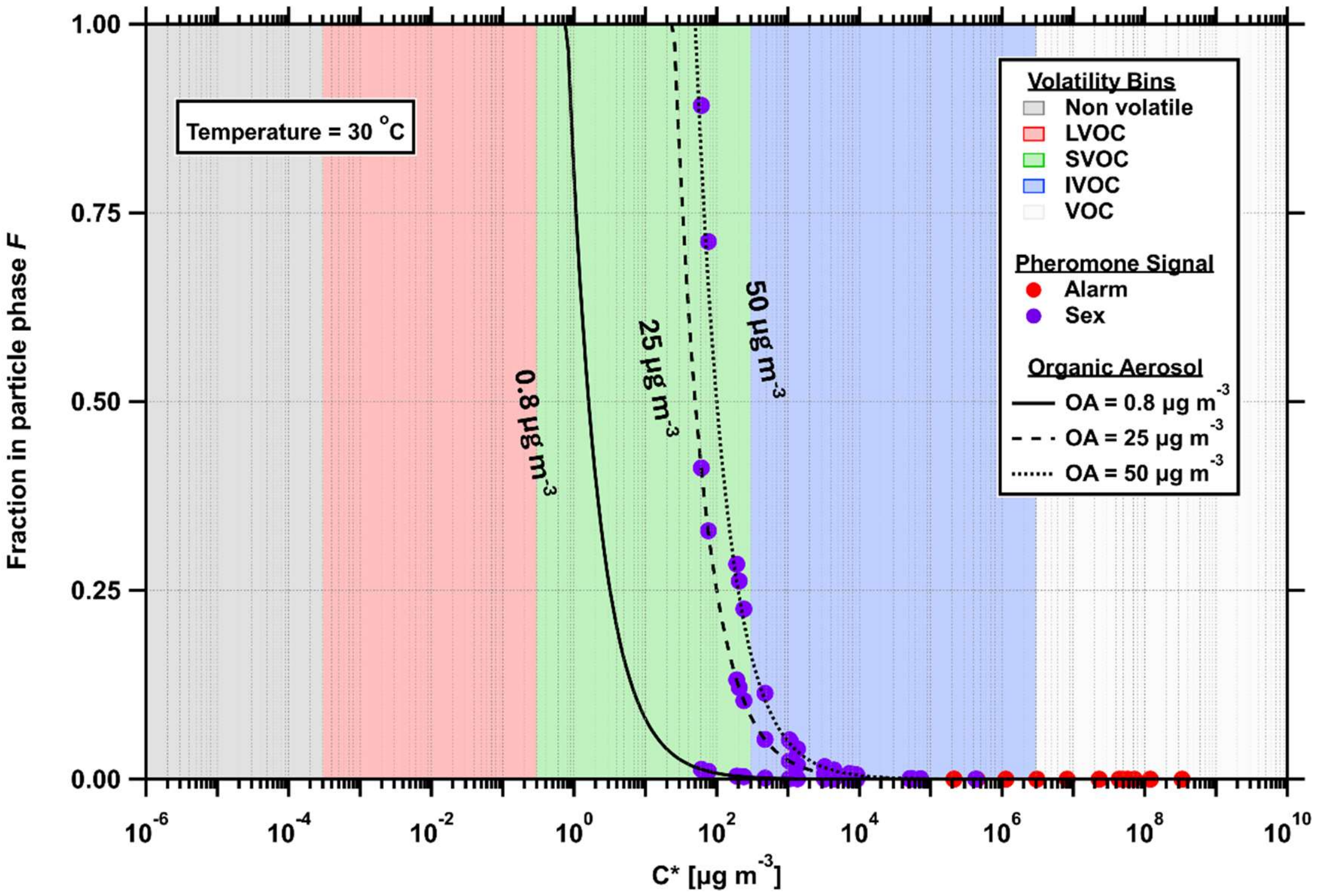

Figure S6 Common alarm and sex pheromones mapped in volatility basis set (VBS) space, with the fraction partitioned into the aerosol phase calculated using the irreversible uptake model (eq. 12) and shown on the y-axis. Results are presented for a temperature of 30 °C and for aerosol mass loadings of 0.8, 25, and 50 µg m^{-3}.

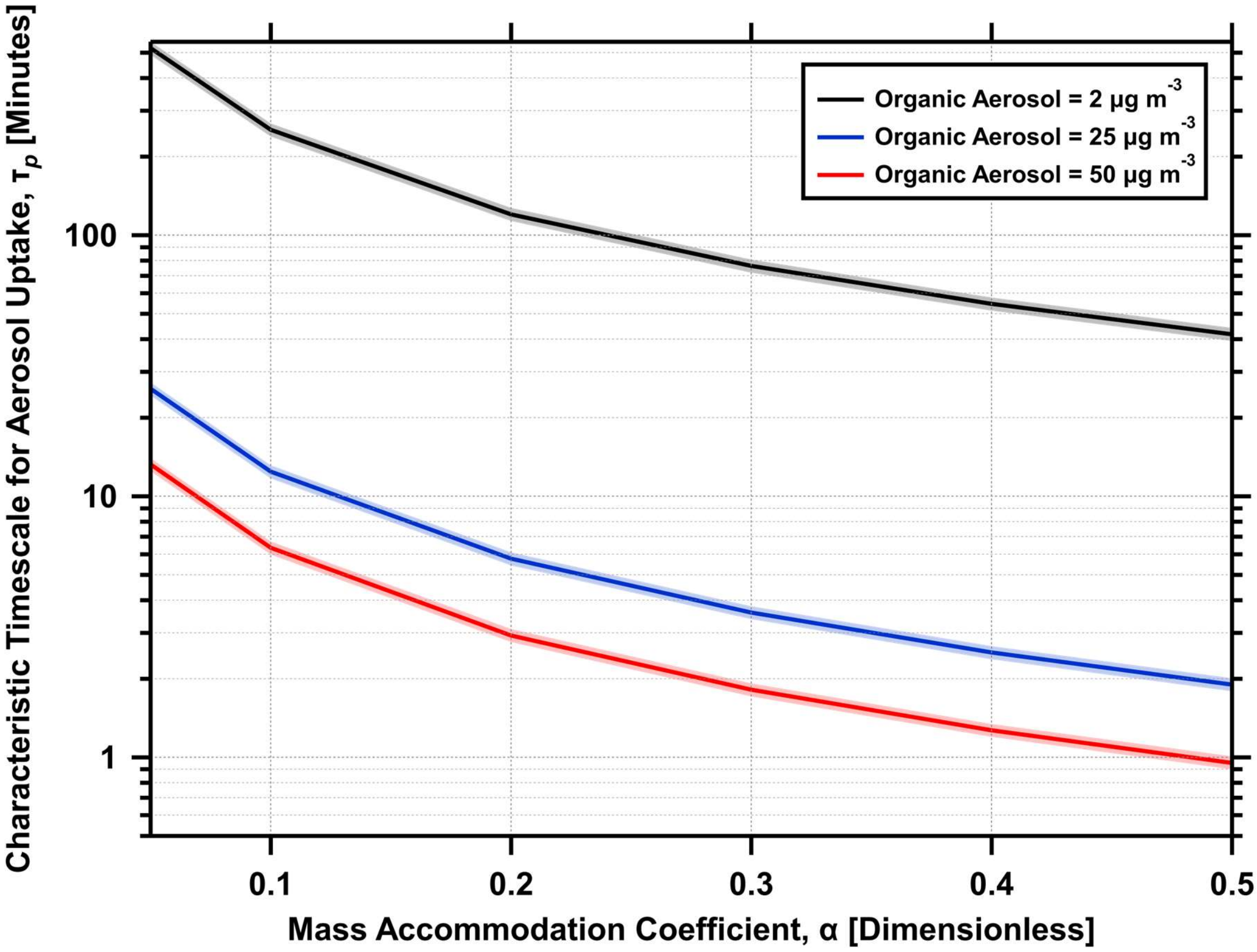

Figure S7. Sensitivity of aerosol uptake timescale (τ_p) to the mass accommodation coefficient (α) for sex pheromones under varying organic aerosol loadings. The x-axis shows α values ranging from 0.05 to 0.5, while the y-axis indicates the average characteristic uptake time. Traces represent different atmospheric organic aerosol concentrations: black (2 µg m^{-3}), blue (25 µg m^{-3}), and red (50 µg m^{-3}). Shaded regions denote ±1 standard deviation across the set of pheromones analysed.

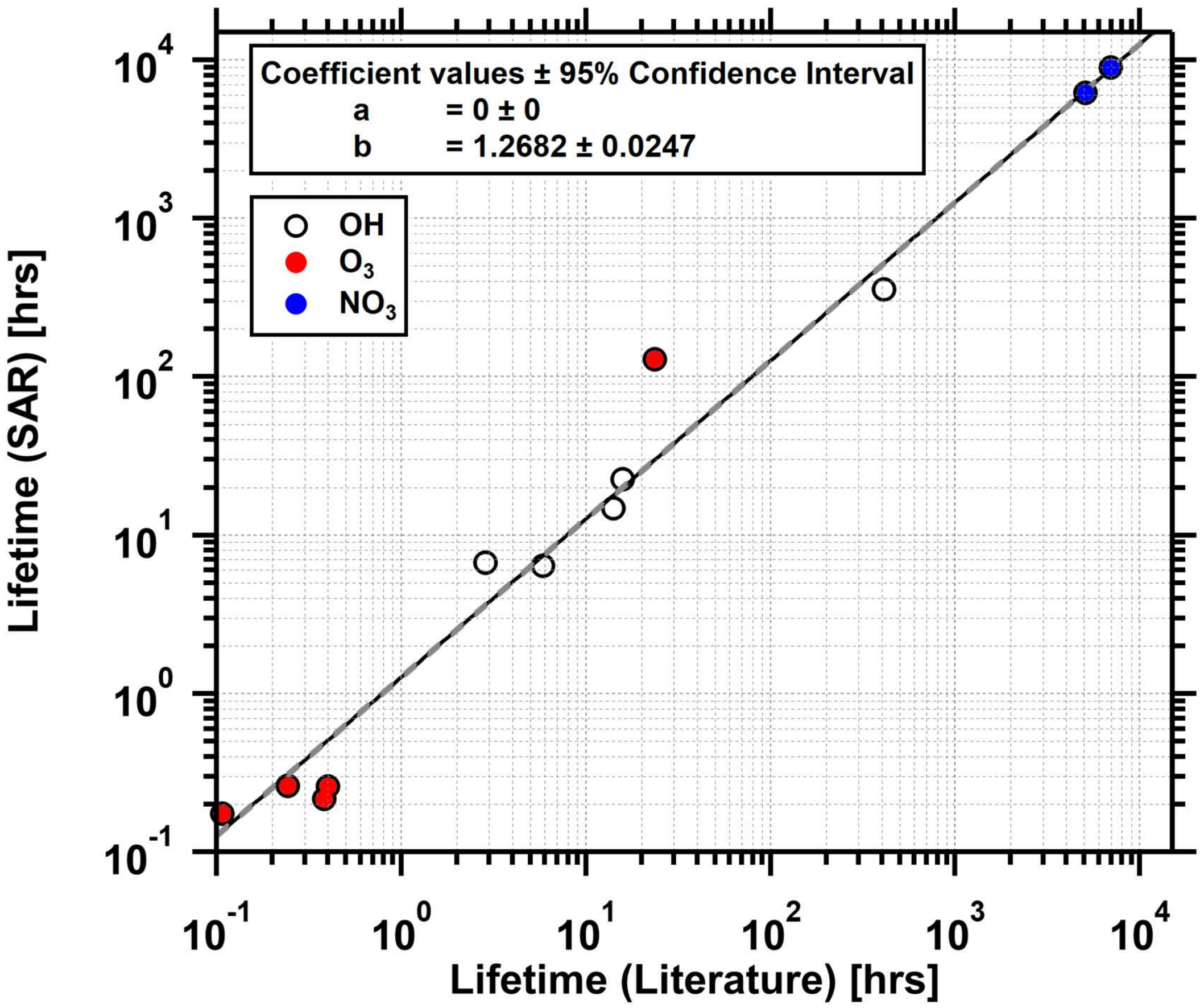

Figure S8. Comparison of SAR-derived atmospheric lifetimes with literature-based lifetimes for OH, O_3 and NO_3 oxidation.

Supplementary Tables

Table S1. Summary of selected insect pheromones and their key physicochemical properties. For each compound, we list the associated insect(s), literature reference, signal type (e.g., alarm or sex), molecular weight (Mw), chemical formula, saturation vapour pressure (SVP), saturation concentration (*C), and pseudo-first-order rate constants for gas-phase reactions with OH, O_3, and NO_3 radicals. Bold values indicate rate constants obtained from literature – all others were derived from structural activity relationships. SVPs obtained from EPI Suite.**

Pheromone	Insect(s)	Ref	Signal Type	M_w [g mol]	Formula	SVP [Pa]	C^* [μg m^{-3}]	k_{OH}	k_{O3}	k_{NO3}
Formic Acid	formicine ant F. rufa	2	Alarm	46.03	CH_2O_2	5030	9.34E+07	**4.50E-13**	1.00E-20	**1.00E-18**
2-Methyl-3-buten-2-ol	Horent, Vespa Crabro	3	Alarm	88.15	$C_5H_{10}O$	7180	2.55E+08	**6.48E-11**	9.55E-18	1.20E-14
Hexanal	Leaf-footed bug, Leptoglossus zonatus (Heteroptera: Coreidae) and	4	Alarm	100.159	$C_6H_{12}O$	1370	5.54E+07	**3.17E-11**	1.00E-20	**1.60E-14**
2-Heptanone	Honeybees	5	Alarm	114.19	$C_7H_{14}O$	709	3.27E+07	**1.17E-11**	1.00E-20	9.78E-17
3-Octanone	Fungus growing ants (Attini)	6	Alarm	128.21	$C_8H_{16}O$	339	1.75E+07	9.30E-12	1.00E-20	1.15E-16
4-Methyl-3-heptanone	Clonal Raider Ant (Ooceraea biroi)	7	Alarm	128.21	$C_8H_{16}O$	725	3.75E+07	9.35E-12	1.00E-20	1.57E-16
Isoamyl acetate	Honeybees	8	Alarm	130.19	$C_7H_{14}O_2$	818	4.30E+07	6.01E-12	1.00E-20	1.78E-16
Citral	Acarid mites	9,10	Alarm/Sex	152.24	$C_{10}H_{16}O$	12.9	7.92E+05	1.36E-10	4.73E-16	9.62E-12
Geraniol	Sycamore lace bugs (Corythuca ciliata); Chrsanthemum Lace Bug (Corythucha marmorata); Ambrosia Beetle (Platypus koryoensis)	11-13	Alarm	154.25	$C_{10}H_{18}O$	2.28	1.42E+05	1.80E-10	9.30E-16	1.57E-11

Decanal	Giant Asian honey bee (Apis dorsata)	[14,15]	Alarm	156.27	$C_{10}H_{20}O$	33.88	2.14E+06	3.45E-11	1.00E-20	**2.20E-14**
Undecane	Silvery field ant (Formica argentea)	[16]	Alarm	156.31	$C_{11}H_{24}$	90.75	5.72E+06	**1.17E-11**	1.00E-20	2.41E-16
(8Z)-8-Dodecen-1-ol	Oriental peach moth (Grapholita molesta)	[17]	Sex	184.32	$C_{12}H_{24}O$	0.06	4.46E+03	7.12E-11	1.20E-16	4.50E-13
(E)-β-Farnesene	Aphids	[18]	Alarm	204.35	$C_{15}H_{24}$	3.33	2.75E+05	3.20E-10	**5.64E-16**	2.33E-11
(E,E)-α-Farnesene	Lesser Mealworm (Alphitobius diaperinus); Termite (Prorhinotermes canalifrons)	[19,20]	Alarm/Sex	204.35	$C_{15}H_{24}$	3.33	2.75E+05	3.20E-10	**5.90E-16**	1.00E-20
(Z)-9-Tetradecenal	*Helicoverpa armigera*	[21]	Sex	210.37	$C_{14}H_{26}O$	0.365	3.10E+04	1.01E-10	1.20E-16	4.56E-13
Farnesol	Twospotted Spider Mite, *Tetranychus urticae*	[22]	Sex	222.37	$C_{15}H_{26}O$	0.00317	2.84E+02	2.69E-10	**2.10E-15**	2.67E-11
(7E,9E)-7,9-Dodecadien-1-yl-acetate	European grapevine moth (Lobesia botrana)	[17]	Sex	224.34	$C_{14}H_{24}O_2$	0.36000	3.26E+04	1.52E-10	3.40E-16	1.41E-11
(8E,10E)-8,10-Dodecadien-1-yl acetate	Chestnut tortrix (Cydia splendana)	[17]	Sex	224.34	$C_{14}H_{24}O_2$	0.36000	3.26E+04	1.52E-10	3.40E-16	1.32E-11
(8Z)-8-Dodecen-1-yl acetate	Oriental peach moth (Grapholita molesta)	[17]	Sex	226.35	$C_{14}H_{26}O_2$	0.47000	4.29E+04	6.90E-11	1.20E-16	4.50E-13
(8E)-8-Dodecen-1-yl acetate	Oriental peach moth (Grapholita molesta)	[17]	Sex	226.35	$C_{14}H_{26}O_2$	0.47000	4.29E+04	6.90E-11	1.70E-16	4.50E-13
(10E,12Z)-Hexadeca-10,12-dienal (Bombykal)	Silk moth (Bombyx mori)	[17]	Sex	236.39	$C_{16}H_{28}O$	0.03000	2.86E+03	1.79E-10	3.40E-16	1.41E-11

(10Z,12E)-Hexadeca-10,12-dien-1-OL (Bombykol)	Silk moth (Bombyx mori)	17	Sex	238.39	$C_{16}H_{30}O$	0.00036	3.44E+01	1.60E-10	3.40E-16	1.41E-11
(Z)-11-Hexadecenal	*Chilo suppressalis*	23	Sex	238.41	$C_{16}H_{30}O$	2.52000	2.42E+05	1.04E-10	1.20E-16	4.56E-13
(E,E)-10,12-hexadecadien-1-ol	*Hemileuca electra*	24	Sex	238.41	$C_{16}H_{30}O$	0.00036	3.44E+01	1.60E-10	3.40E-16	1.41E-11
(11Z)-11-Hexadecenal	Box tree moth (Cydolima perspectalis)	17	Sex	238.41	$C_{16}H_{30}O$	2.52000	2.42E+05	9.60E-11	1.20E-16	4.56E-13
(Z)-11-Hexadecen-1-ol	Lepidoptera: Noctuidae	25	Sex	240.41	$C_{16}H_{32}O$	0.00038	3.70E+01	8.45E-11	1.20E-16	4.50E-13
(3E,8Z,11Z)-3,8,11-Tetradecatrien-1-yl acetate	South American tomato moth (Tuta absoluta)	17	Sex	250.38	$C_{16}H_{26}O_2$	0.04000	4.04E+03	1.78E-10	4.11E-16	1.35E-12
(9Z)-9-Tetradecen-1-yl acetate	Fall army moth (Spodoptera frugiperda)	17	Sex	254.41	$C_{16}H_{30}O_2$	0.05000	5.13E+03	7.18E-11	1.20E-16	4.50E-13
(Z)-11-Octadecenal	Wax moth (Achroia grisella)	26	Sex	266.45	$C_{18}H_{34}O$	0.00524	5.63E+02	1.06E-10	1.20E-16	4.56E-13
(Z)-11-Octadecen-1-ol	Rice leaffolder (Cnaphalocrocis medinalis, Lepidoptera: Crambidae)	27	Sex	268.45	$C_{18}H_{36}O$	0.00100	1.08E+02	8.73E-11	1.20E-16	4.51E-13
(13Z)-13-Hexadecen-11-yn-1-yl acetate	Pine processionary moth (Thaumetopoea pityocampa)	17	Sex	278.4	$C_{18}H_{30}O_2$	0.00112	1.26E+02	1.12E-10	5.72E-18	1.00E-20
(Z)-11-Hexadecenyl acetate	Diamondback moth (Lepidoptera: Plutellidae)	28	Sex	282.45	$C_{18}H_{34}O_2$	0.01510	1.72E+03	8.22E-11	1.20E-16	4.50E-13
(Z)-11-Hexadecenyl propionate	Thaumetopoea pityocampa	29	Sex	296.47	$C_{19}H_{36}O_2$	0.00690	8.25E+02	7.47E-11	1.20E-16	4.50E-13

	(synthetic analog tested)									
(Z)-11-Octadecenyl acetate	Lonfhorned beetle (Ortholeptura valida)	[30]	Sex	310.5	$C_{20}H_{38}O_2$	0.00075	9.42E+01	7.74E-11	1.20E-16	4.50E-13
Z-9-Tricosene	Olive fruit fly (Bactrocera oleae Housefly (Musca domestica)	[31]	Sex	322.62	$C_{23}H_{46}$	0.00396	5.15E+02	8.25E-11	1.20E-16	4.50E-13

Table S2 Pheromone lifetimes (hours) calculated for a range of pollution scenarios and ambient temperatures. All values were calculated using the reversible uptake model (eq. 5).

		Pristine				Polluted				Highly Polluted			
		Equator		Mid Latitudes		Equator		Mid Latitudes		Equator		Mid Latitudes	
**Pheromone**	**Type**	**Day (30 C)**	**Night (25 C)**	**Day (25 C)**	**Night (15 C)**	**Day (30 C)**	**Night (25 C)**	**Day (25 C)**	**Night (15 C)**	**Day (30 C)**	**Night (25 C)**	**Day (25 C)**	**Night (15 C)**
Formic Acid	Alarm	410.05	34808.79	410.03	34623.58	404.28	21530.37	404.16	21179.97	397.28	14578.99	397.05	14259.50
2-Methyl-3-buten-2-ol	Alarm	2.79	126.13	2.79	126.97	2.55	40.49	2.55	39.32	2.31	21.79	2.30	21.11
Hexanal	Alarm	5.84	582.18	5.84	582.13	5.84	553.29	5.84	553.05	5.84	526.29	5.84	525.87
2-Heptanone	Alarm	15.83	1571.74	15.83	1571.36	15.82	1528.14	15.82	1526.35	15.81	1477.18	15.81	1473.84
3-Octanone	Alarm	19.90	1973.35	19.90	1972.75	19.89	1904.82	19.89	1902.04	19.87	1826.04	19.87	1820.93
4-Methyl-3-heptanone	Alarm	19.81	1963.86	19.81	1963.26	19.79	1895.30	19.79	1892.54	19.78	1816.67	19.78	1811.62
Isoamyl acetate	Alarm	30.78	3037.61	30.78	3036.20	30.75	2875.88	30.75	2869.53	30.71	2697.99	30.71	2686.84
Citral	Alarm/Sex	0.87	4.59	0.87	4.45	0.36	0.91	0.35	0.88	0.21	0.46	0.20	0.44
Geraniol	Alarm	0.56	2.36	0.56	2.29	0.20	0.47	0.20	0.45	0.11	0.23	0.11	0.23
Decanal	Alarm	5.37	534.93	5.37	534.88	5.37	503.49	5.37	503.29	5.37	474.98	5.36	474.62
Undecane	Alarm	14.03	1394.21	14.03	1393.91	14.02	1358.60	14.02	1357.18	14.01	1317.07	14.01	1314.40
(8Z)-8-Dodecen-1-ol	Sex	2.04	17.53	2.04	16.98	1.10	3.66	1.09	3.49	0.69	1.83	0.68	1.72
(E)-β-Farnesene	Alarm	0.45	3.72	0.45	3.60	0.24	0.73	0.24	0.71	0.15	0.37	0.15	0.36
(E,E)-α-Farnesene	Alarm/Sex	0.45	3.59	0.44	3.48	0.23	0.76	0.23	0.73	0.15	0.38	0.14	0.37
(Z)-9-Tetradecenal	Sex	1.54	17.06	1.54	16.54	0.94	3.66	0.93	3.53	0.63	1.85	0.62	1.78
Farnesol	Sex	0.31	1.05	0.30	1.02	0.09	0.19	0.09	0.17	0.05	0.09	0.04	0.07

(7E,9E)-7,9-Dodecadien-1-yl-acetate	Sex	0.90	6.25	0.89	6.05	0.43	1.21	0.43	1.17	0.26	0.61	0.26	0.59
(8E,10E)-8,10-Dodecadien-1-yl acetate	Sex	0.90	6.25	0.89	6.05	0.43	1.22	0.43	1.18	0.26	0.61	0.26	0.59
(8Z)-8-Dodecen-1-yl acetate	Sex	2.10	17.57	2.09	17.02	1.12	3.68	1.11	3.56	0.70	1.85	0.70	1.79
(8E)-8-Dodecen-1-yl acetate	Sex	1.92	12.65	1.91	12.25	0.90	2.61	0.89	2.53	0.54	1.31	0.53	1.27
(10E,12Z)-Hexadeca-10,12-dienal (Bombykal)	Sex	0.79	6.19	0.79	5.99	0.41	1.20	0.40	1.15	0.25	0.60	0.25	0.56
(10Z,12E)-Hexadeca-10,12-dien-1-OL (Bombykol)	Sex	0.86	6.11	0.85	5.65	0.31	0.71	0.26	0.37	0.15	0.25	0.11	0.11
(Z)-11-Hexadecenal	Sex	1.51	17.01	1.50	16.50	0.92	3.66	0.92	3.54	0.62	1.85	0.62	1.79
(E,E)-10,12-hexadecadien-1-ol	Sex	0.86	6.11	0.85	5.65	0.31	0.71	0.26	0.37	0.15	0.25	0.11	0.11
(11Z)-11-Hexadecenal	Sex	1.61	17.13	1.60	16.61	0.96	3.66	0.95	3.54	0.64	1.85	0.63	1.79
(Z)-11-Hexadecen-1-ol	Sex	1.77	16.97	1.75	15.33	0.78	2.20	0.62	0.89	0.40	0.76	0.28	0.24
(3E,8Z,11Z)-3,8,11-Tetradecatrien-1-yl acetate	Sex	0.76	5.21	0.75	5.05	0.36	1.07	0.36	1.02	0.22	0.54	0.21	0.50

(9Z)-9-Tetradecen-1-yl acetate	Sex	2.03	17.52	2.02	16.97	1.10	3.66	1.08	3.50	0.69	1.83	0.68	1.73
(Z)-11-Octadecenal	Sex	1.47	16.95	1.47	16.37	0.89	3.50	0.87	3.02	0.59	1.69	0.56	1.30
(Z)-11-Octadecen-1-ol	Sex	1.73	17.15	1.72	16.29	0.90	2.98	0.82	1.87	0.53	1.24	0.44	0.61
(13Z)-13-Hexadecen-11-yn-1-yl acetate	Sex	1.64	115.81	1.63	112.59	1.44	44.56	1.33	29.33	1.25	22.27	1.07	11.53
(Z)-11-Hexadecenyl acetate	Sex	1.82	17.35	1.82	16.79	1.03	3.62	1.01	3.37	0.66	1.80	0.64	1.60
(Z)-11-Hexadecenyl propionate	Sex	1.97	17.46	1.96	16.89	1.06	3.57	1.04	3.28	0.66	1.74	0.64	1.51
(Z)-11-Octadecenyl acetate	Sex	1.91	17.29	1.90	16.31	0.94	2.91	0.84	1.66	0.54	1.18	0.43	0.52
Z-9-Tricosene	Sex	1.82	17.33	1.81	16.70	1.01	3.50	0.98	2.93	0.64	1.68	0.60	1.23

Table S3 Pheromone lifetimes calculated for a range of pollution scenarios and ambient temperatures. All values were calculated using the irreversible uptake model (eq. 12).

		Pristine				Polluted				Highly Polluted			

		Equator		Mid Latitudes		Equator		Mid Latitudes		Equator		Mid Latitudes	
**Pheromone**	**Type**	**Day (30 C)**	**Night (25 C)**	**Day (25 C)**	**Night (15 C)**	**Day (30 C)**	**Night (25 C)**	**Day (25 C)**	**Night (15 C)**	**Day (30 C)**	**Night (25 C)**	**Day (25 C)**	**Night (15 C)**
Formic Acid	Alarm	410.05	34808.79	410.03	34623.58	404.28	21530.37	404.16	21179.97	397.28	14578.99	397.05	14259.50
2-Methyl-3-buten-2-ol	Alarm	2.79	126.13	2.79	123.73	2.54	38.92	2.54	37.79	2.29	20.88	2.28	20.23
Hexanal	Alarm	5.84	582.18	5.84	582.13	5.84	553.29	5.84	553.05	5.84	526.29	5.84	525.87
2-Heptanone	Alarm	15.83	1571.74	15.83	1571.36	15.82	1528.14	15.82	1526.35	15.81	1477.18	15.81	1473.84
3-Octanone	Alarm	19.90	1973.352	19.904	1972.753	19.891	1904.822	19.890	1902.036	19.873	1826.041	19.873	1820.926
4-Methyl-3-heptanone	Alarm	19.81	1963.857	19.808	1963.265	19.794	1895.296	19.794	1892.539	19.777	1816.675	19.777	1811.615
Isoamyl acetate	Alarm	30.78	3037.614	30.783	3036.197	30.751	2875.877	30.750	2869.534	30.709	2697.987	30.708	2686.844
Citral	Alarm/Sex	0.87	4.594	0.867	4.446	0.358	0.910	0.353	0.881	0.206	0.457	0.203	0.442
Geraniol	Alarm	0.56	2.364	0.557	2.287	0.199	0.467	0.196	0.452	0.110	0.234	0.109	0.226
Decanal	Alarm	5.37	534.928	5.367	534.884	5.366	503.488	5.366	503.286	5.365	474.983	5.365	474.623
Undecane	Alarm	14.03	1394.21	14.03	1393.91	14.02	1358.60	14.02	1357.18	14.01	1317.07	14.01	1314.40
(8Z)-8-Dodecen-1-ol	Sex	2.04	17.533	2.037	16.977	1.099	3.661	1.086	3.492	0.695	1.832	0.683	1.718
(E)-β-Farnesene	Alarm	0.45	3.716	0.449	3.600	0.239	0.728	0.237	0.706	0.151	0.367	0.149	0.356
(E,E)-α-Farnesene	Alarm/Sex	0.45	3.590	0.445	3.477	0.233	0.755	0.230	0.730	0.146	0.380	0.144	0.367
(Z)-9-Tetradecenal	Sex	1.54	17.058	1.537	16.537	0.937	3.657	0.929	3.532	0.628	1.845	0.621	1.779
Farnesol	Sex	0.31	1.055	0.303	1.018	0.091	0.193	0.087	0.157	0.047	0.087	0.043	0.052
(7E,9E)-7,9-Dodecadien-1-yl-acetate	Sex	0.90	6.248	0.891	6.051	0.434	1.210	0.430	1.171	0.264	0.608	0.261	0.588

(8E,10E)-8,10-Dodecadien-1-yl acetate	Sex	0.90	6.252	0.891	6.054	0.434	1.216	0.430	1.177	0.264	0.612	0.261	0.591
(8Z)-8-Dodecen-1-yl acetate	Sex	2.10	17.573	2.088	17.020	1.117	3.681	1.106	3.556	0.705	1.852	0.696	1.787
(8E)-8-Dodecen-1-yl acetate	Sex	1.92	12.650	1.911	12.245	0.898	2.615	0.888	2.526	0.539	1.313	0.532	1.266
(10E,12Z)-Hexadeca-10,12-dienal (Bombykal)	Sex	0.79	6.191	0.789	5.994	0.406	1.199	0.401	1.147	0.252	0.598	0.247	0.563
(10Z,12E)-Hexadeca-10,12-dien-1-OL (Bombykol)	Sex	0.86	6.103	0.854	5.625	0.263	0.351	0.137	0.042	0.042	0.021	0.018	0.021
(Z)-11-Hexadecenal	Sex	1.51	17.014	1.502	16.496	0.924	3.657	0.917	3.537	0.623	1.848	0.616	1.786
(E,E)-10,12-hexadecadien-1-ol	Sex	0.86	6.103	0.854	5.625	0.263	0.351	0.137	0.042	0.042	0.021	0.018	0.021
(11Z)-11-Hexadecenal	Sex	1.61	17.134	1.601	16.609	0.961	3.663	0.952	3.542	0.639	1.849	0.632	1.787
(Z)-11-Hexadecen-1-ol	Sex	1.77	16.960	1.754	15.192	0.695	1.201	0.346	0.066	0.203	0.034	0.022	0.033
(3E,8Z,11Z)-3,8,11-Tetradecatrien-1-yl acetate	Sex	0.76	5.215	0.755	5.046	0.362	1.074	0.358	1.023	0.219	0.536	0.215	0.502
(9Z)-9-Tetradecen-1-yl acetate	Sex	2.03	17.524	2.024	16.969	1.096	3.664	1.083	3.502	0.694	1.835	0.682	1.729
(Z)-11-Octadecenal	Sex	1.47	16.947	1.467	16.368	0.891	3.494	0.865	2.925	0.587	1.671	0.553	1.118

(Z)-11-Octadecen-1-ol	Sex	1.73	17.151	1.723	16.277	0.888	2.826	0.773	0.351	0.490	0.930	0.333	0.034
(13Z)-13-Hexadecen-11-yn-1-yl acetate	Sex	1.64	115.805	1.634	112.516	1.426	42.792	1.277	11.444	1.187	18.130	0.875	0.326
(Z)-11-Hexadecenyl acetate	Sex	1.82	17.348	1.817	16.788	1.027	3.621	1.011	3.355	0.661	1.794	0.643	1.575
(Z)-11-Hexadecenyl propionate	Sex	1.97	17.462	1.961	16.888	1.061	3.568	1.038	3.253	0.663	1.733	0.638	1.460
(Z)-11-Octadecenyl acetate	Sex	1.91	17.293	1.898	16.291	0.928	2.707	0.782	0.070	0.494	0.796	0.296	0.035
Z-9-Tricosene	Sex	1.82	17.325	1.811	16.704	1.009	3.497	0.976	2.803	0.637	1.659	0.595	0.973

Table S4. Sensitivity of aerosol-phase fraction F to condensed-phase non-ideality (y_0) at 15 °C. Saturation concentrations C^* (µg m⁻³) and resulting aerosol-phase fractions F (%) are shown for each compound under three organic-aerosol loadings: Pristine (0.8 µg m⁻³), Polluted (25 µg m⁻³), and Highly Polluted (50 µg m⁻³). F is calculated at 15 °C using F=1/(1+C^*/M), with C^* scaled by the activity coefficient y_0 (1, 3, 5, 10). Larger y_0 values increase C^* and reduce F, illustrating the influence of non-ideality on partitioning for low-volatility pheromones.

**Compound**	**Scenario**	**C^* [µg m^{-3}]**	**F (y_0 = 1) [%]**	**F (y_0 = 3) [%]**	**F (y_0 = 5) [%]**	**F (y_0 = 10) [%]**
(Z)-11-Hexadecen-1-ol	Pristine (0.8 µg m^{-3})	8.05	0.09	0.03	0.02	0.01
	Polluted (25 µg m^{-3})	8.05	0.76	0.51	0.38	0.24
	Highly Polluted (50 µg m^{-3})	8.05	0.86	0.67	0.55	0.38
(10Z,12E)-Hexadeca-10,12-dien-1-OL (Bombykol)	Pristine (0.8 µg m^{-3})	10.39	0.07	0.03	0.02	0.01
	Polluted (25 µg m^{-3})	10.39	0.71	0.45	0.32	0.19
	Highly Polluted (50 µg m^{-3})	10.39	0.83	0.62	0.49	0.32
(E,E)-10,12-hexadecadien-1-ol	Pristine (0.8 µg m^{-3})	10.39	0.07	0.03	0.02	0.01
	Polluted (25 µg m^{-3})	10.39	0.71	0.45	0.32	0.19
	Highly Polluted (50 µg m^{-3})	10.39	0.83	0.62	0.49	0.32
(Z)-11-Octadecenyl acetate	Pristine (0.8 µg m^{-3})	21.37	0.04	0.01	0.01	0.00
	Polluted (25 µg m^{-3})	21.37	0.54	0.28	0.19	0.10
	Highly Polluted (50 µg m^{-3})	21.37	0.70	0.44	0.32	0.19
(Z)-11-Octadecen-1-ol	Pristine (0.8 µg m^{-3})	27.58	0.03	0.01	0.01	0.00

	Polluted (25 μg m^{-3})	27.58	0.48	0.23	0.15	0.08
	Highly Polluted (50 μg m^{-3})	27.58	0.64	0.38	0.27	0.15
(13Z)-13-Hexadecen-11-yn-1-yl acetate	Pristine (0.8 μg m^{-3})	32.09	0.02	0.01	0.00	0.00
	Polluted (25 μg m^{-3})	32.09	0.44	0.21	0.13	0.07
	Highly Polluted (50 μg m^{-3})	32.09	0.61	0.34	0.24	0.13

Supplementary References

1 Shiraiwa, M. & Pöschl, U. Mass accommodation and gas–particle partitioning in secondary organic aerosols: dependence on diffusivity, volatility, particle-phase reactions, and penetration depth. *Atmos. Chem. Phys.* **21**, 1565-1580 (2021). https://doi.org/10.5194/acp-21-1565-2021

2 Lofqvist, J. FORMIC-ACID AND SATURATED-HYDROCARBONS AS ALARM PHEROMONES FOR ANT FORMICA-RUFA. *Journal of Insect Physiology* **22**, 1331-1346 (1976). https://doi.org/10.1016/0022-1910(76)90155-4

3 MacLean, C. & Schmolz, E. Calorimetric investigations on the action of alarm pheromones in the hornet Vespa crabro. *Thermochimica Acta* **414**, 71-77 (2004). https://doi.org/https://doi.org/10.1016/j.tca.2003.11.016

4 Soares Leal, W., Ricardo Panizzi, A. & Carla Niva, C. Alarm pheromone system of leaf-footed bugLeptoglossus zonatus (Heteroptera: Coreidae). *Journal of Chemical Ecology* **20**, 1209-1216 (1994). https://doi.org/10.1007/BF02059755

5 Papachristoforou, A. *et al.* The bite of the honeybee: 2-heptanone secreted from honeybee mandibles during a bite acts as a local anaesthetic in insects and mammals. *PLoS One* **7**, e47432 (2012). https://doi.org/10.1371/journal.pone.0047432

6 Norman, V. C., Butterfield, T., Drijfhout, F., Tasman, K. & Hughes, W. O. Alarm Pheromone Composition and Behavioral Activity in Fungus-Growing Ants. *J Chem Ecol* **43**, 225-235 (2017). https://doi.org/10.1007/s10886-017-0821-4

7 Lopes, L. E., Frank, E. T., Kárpáti, Z., Schmitt, T. & Kronauer, D. J. C. The Alarm Pheromone and Alarm Response of the Clonal Raider Ant. *J Chem Ecol* **49**, 1-10 (2023). https://doi.org/10.1007/s10886-023-01407-4

8 Gong, Z. *et al.* High Concentrations of the Alarm Pheromone Component, Isopentyl Acetate, Reduces Foraging and Dancing in Apis mellifera Ligustica and Apis cerana Cerana. *Journal of Insect Behavior* **30**, 188-198 (2017). https://doi.org/10.1007/s10905-017-9606-4

9 Kuwahara, Y., Matsumoto, K. & Wada, Y. Pheromone study on acarid mites IV : Citral : composition and function as an alarm pheromone and its secretory gland in four species of acarid mites. *Medical Entomology and Zoology* **31**, 73-80 (1980). https://doi.org/10.7601/mez.31.73

10 Larsdotter-Mellström, H. *et al.* It's All in the Mix: Blend-Specific Behavioral Response to a Sexual Pheromone in a Butterfly. *Frontiers in Physiology* **Volume 7 - 2016** (2016). https://doi.org/10.3389/fphys.2016.00068

11 Kuwahara, Y., Kawai, A., Shimizu, N., Tokumaru, S. & Ueyama, H. Geraniol, E-3,7-dimethyl-2,6-octadien-1-ol, as the Alarm Pheromone of the Sycamore Lace Bug Corythucha ciliata (Say). *Journal of Chemical Ecology* **37**, 1211-1215 (2011). https://doi.org/10.1007/s10886-011-0025-2

12 Watanabe, K. & Shimizu, N. Alarm Pheromone Activity of Nymph-specific Geraniol in Chrysanthemum Lace Bug Corythucha marmorata against Adults and Nymphs. *Nat Prod Commun* **10**, 1495-1498 (2015).

13 Kim, J., Lee, S.-G., Shin, S.-C., Kwon, Y.-D. & Park, I.-K. Male-Produced Aggregation Pheromone Blend in Platypus koryoensis. *Journal of Agricultural and Food Chemistry* **57**, 1406-1412 (2009). https://doi.org/10.1021/jf8032717

14 Kwadha, C. A. *et al.* Decanal as a major component of larval aggregation pheromone of the greater wax moth, Galleria mellonella. *Journal of Applied Entomology* **143**, 417-429 (2019). https://doi.org/https://doi.org/10.1111/jen.12617

15 Li, J., Wang, Z., Tan, K., Qu, Y. & Nieh, J. C. Effects of natural and synthetic alarm pheromone and individual pheromone components on foraging behavior of the giant Asian honey bee, Apis dorsata. *J Exp Biol* **217**, 3512-3518 (2014). https://doi.org/10.1242/jeb.110171

16 Lenz, E. L., Krasnec, M. O. & Breed, M. D. Identification of Undecane as an Alarm Pheromone of the Ant Formica argentea. *Journal of Insect Behavior* **26**, 101-108 (2013). https://doi.org/10.1007/s10905-012-9337-5

17 Tiryaeva, A. *et al.* Estimating surface tension of moth sex pheromones. *Chemoecology* **35**, 1-9 (2025). https://doi.org/10.1007/s00049-024-00412-7

18 Kunert, G., Reinhold, C. & Gershenzon, J. Constitutive emission of the aphid alarm pheromone, (E)-β-farnesene, from plants does not serve as a direct defense against aphids. *BMC Ecology* **10**, 23 (2010). https://doi.org/10.1186/1472-6785-10-23

19 Hassemer, M. J. *et al.* Revisiting the Male-Produced Aggregation Pheromone of the Lesser Mealworm, Alphitobius diaperinus (Coleoptera, Tenebrionidae): Identification of a Six-Component Pheromone from a Brazilian Population. *Journal of Agricultural and Food Chemistry* **64**, 6809-6818 (2016). https://doi.org/10.1021/acs.jafc.6b02235

20 Šobotník, J. *et al.* (E,E)-α-Farnesene, an Alarm Pheromone of the Termite Prorhinotermes canalifrons. *Journal of Chemical Ecology* **34**, 478-486 (2008). https://doi.org/10.1007/s10886-008-9450-2

21 Zhang, J.-P., Salcedo, C., Fang, Y.-L., Zhang, R.-J. & Zhang, Z.-N. An overlooked component: (Z)-9-tetradecenal as a sex pheromone in Helicoverpa armigera. *Journal of Insect Physiology* **58**, 1209-1216 (2012). https://doi.org/https://doi.org/10.1016/j.jinsphys.2012.05.018

22 Regev, S. & Cone, W. W. Evidence of Farnesol as a Male Sex Attractant of the Twospotted Spider Mite, Tetranychus urticae Koch (Acarina: Tetranychidae) 1. *Environmental Entomology* **4**, 307-311 (1975). https://doi.org/10.1093/ee/4.2.307

23 Nesbitt, B. F., Beevor, P. S., Hall, D. R., Lester, R. & Dyck, V. A. Identification of the female sex pheromones of the moth, Chilo suppressalis. *Journal of Insect Physiology* **21**, 1883-1886 (1975). https://doi.org/https://doi.org/10.1016/0022-1910(75)90218-8

24 McElfresh, J. S. & Millar, J. G. Geographic Variation in Sex Pheromone Blend of Hemileuca electra from Southern California. *Journal of Chemical Ecology* **25**, 2505-2525 (1999). https://doi.org/10.1023/A:1020826208715

25 Heath, R. R., Mitchell, E. R. & Cibrian Tovar, J. Effect of release rate and ratio of (Z)-11-hexadecen-1-ol from synthetic pheromone blends on trap capture ofHeliothis subflexa (Lepidoptera: Noctuidae). *J Chem Ecol* **16**, 1259-1268 (1990). https://doi.org/10.1007/bf01021024

26 Grigor'eva, N. Y., Tsiklauri, P. G. & Buevich, A. V. Synthesis ofZ-11-octadecenal, the sex pheromone of the wax moth. *Russian Chemical Bulletin* **43**, 1079-1080 (1994). https://doi.org/10.1007/BF01558085

27 Kawazu, K. *et al.* Sex pheromone components of the rice leaffolder, Cnaphalocrocis medinalis (Lepidoptera: Crambidae), in Indonesia. *Journal of Asia-Pacific Entomology* **12**, 97-99 (2009). https://doi.org/https://doi.org/10.1016/j.aspen.2009.01.001

28 Tamaki, Y. *et al.* (Z)-11-Hexadecenal and (Z)-11-Hexadecenyl Acetate : Sex-Pheromone Components of the Diamondback Moth (Lepidoptera : Plutellidae). *Applied Entomology and Zoology* **12**, 208-210 (1977). https://doi.org/10.1303/aez.12.208

29 Camps, F. *et al.* Analogs of sex pheromone of processionary moth,Thaumetopoea pityocampa: Synthesis and biological activity. *Journal of Chemical Ecology* **14**, 1331-1346 (1988). https://doi.org/10.1007/BF01020138

30 Ray, A. M. *et al.* cis-Vaccenyl acetate, a female-produced sex pheromone component of Ortholeptura valida, a longhorned beetle in the subfamily Lepturinae. *J Chem Ecol* **37**, 173-178 (2011). https://doi.org/10.1007/s10886-011-9908-5

31 Carpita, A. *et al.* (Z)-9-tricosene identified in rectal gland extracts of Bactrocera oleae males: first evidence of a male-produced female attractant in olive fruit fly. *Naturwissenschaften* **99**, 77-81 (2012). https://doi.org/10.1007/s00114-011-0868-y
